# Targeting Replicative Stress and DNA Repair by Combining PARP and Wee1 Kinase Inhibitors Is Synergistic in Triple Negative Breast Cancers with Cyclin E or *BRCA1* Alteration

**DOI:** 10.3390/cancers13071656

**Published:** 2021-04-01

**Authors:** Xian Chen, Dong Yang, Jason P. W. Carey, Cansu Karakas, Constance Albarracin, Aysegul A. Sahin, Banu K. Arun, Merih Guray Durak, Mi Li, Mehrnoosh Kohansal, Tuyen N. Bui, Min-Jin Ha, Kelly K. Hunt, Khandan Keyomarsi

**Affiliations:** 1 Department of Experimental Radiation Oncology, The University of Texas MD Anderson Cancer Center, Houston, TX 77030, USA; Xian.chen@uth.tmc.edu (X.C.); dong.yang@qiagen.com (D.Y.); jcarey@grailbio.com (J.P.W.C.); Cansu.Karakas@rwjbh.org (C.K.); merih.guray@deu.edu.tr (M.G.D.); MLi10@mdanderson.org (M.L.); mkohansa@CougarNet.UH (M.K.); tnbui@mdanderson.org (T.N.B.); 2 Department of Pathology, The University of Texas MD Anderson Cancer Center, Houston, TX 77030, USA; calbarra@mdanderson.org (C.A.); asahin@mdanderson.org (A.A.S.); 3 Department of Breast Medical Oncology, The University of Texas MD Anderson Cancer Center, Houston, TX 77030, USA; barun@mdanderson.org; 4 Department of Bioinformatics, The University of Texas MD Anderson Cancer Center, Houston, TX 77030, USA; MJHa@mdanderson.org; 5 Department of Breast Surgical Oncology, The University of Texas MD Anderson Cancer Center, Houston, TX 77030, USA; khunt@mdanderson.org

**Keywords:** cyclin E, low molecular weight cyclin E (LMWE) PARP, Wee1 kinase, DNA replication stress, BRCA

## Abstract

**Simple Summary:**

Triple-negative breast cancer (TNBC) is a subtype of invasive breast cancer with an aggressive phenotype that has decreased survival compared with other types of breast cancers, due in part to the lack of biomarker driven targeted therapies. Here, we show that breast cancer patients whose tumors show high levels of cyclin E expression have a higher prevalence of *BRCA1/2* alterations and have the worst clinical outcomes. In vitro and in vivo studies revealed that combination therapies with poly (ADP-ribose) polymerase (PARP) and Wee1 kinase inhibitors in TNBC cells with either *BRCA1* mutations or high levels of cyclin E results in synergistic cell death due to induction of replicative stress and downregulation of DNA repair. These studies suggest that by preselecting patients whose tumors have high cyclin E levels or harbor mutations in *BRCA1*, only those cases with the highest replicative stress properties will be subjected to combination treatment and likely result in synergistic activity of the two agents.

**Abstract:**

The identification of biomarker-driven targeted therapies for patients with triple negative breast cancer (TNBC) remains a major clinical challenge, due to a lack of specific targets. Here, we show that cyclin E, a major regulator of G1 to S transition, is deregulated in TNBC and is associated with mutations in DNA repair genes (e.g., *BRCA1/2*). Breast cancers with high levels of cyclin E not only have a higher prevalence of *BRCA1/2* mutations, but also are associated with the worst outcomes. Using several in vitro and in vivo model systems, we show that TNBCs that harbor either mutations in *BRCA1/2* or overexpression of cyclin E are very sensitive to the growth inhibitory effects of AZD-1775 (Wee 1 kinase inhibitor) when used in combination with MK-4837 (PARP inhibitor). Combination treatment of TNBC cell lines with these two agents results in synergistic cell killing due to induction of replicative stress, downregulation of DNA repair and cytokinesis failure that results in increased apoptosis. These findings highlight the potential clinical application of using cyclin E and BRCA mutations as biomarkers to select only those patients with the highest replicative stress properties that may benefit from combination treatment with Wee 1 kinase and PARP inhibitors.

## 1. Introduction

Breast cancer is the most frequent cancer type in women in the United States with 281,550 new cases expected in 2021, representing 30% of all new cancer cases in women [1]. Triple-negative breast cancer (TNBC) is an aggressive subtype of breast cancer with a phenotype demonstrating a lack of estrogen receptor (ER), progesterone receptor (PR) and human epidermal growth factor receptor 2 (HER-2). TNBC accounts for up to 17% of all breast cancers and up to 50% of early-stage TNBCs (stage I to III) develop recurrence with a 37% mortality rate in the first five years after initial diagnosis [2,3,4]. Metastatic TNBC (stage IV) patients have short progression-free survival (median 3 to 4 months) due to failure of first-line chemotherapy [2]. The TNBC survival rates are lower compared to other subtypes of breast cancer, due in part to a lack of targeted therapies. Identification of biomarker-driven targeted therapies for TNBC patients is a major unmet clinical need.

TNBC has been shown to have a higher prevalence of mutations in DNA repair pathways. For example, 10–30% of TNBC patients who are less than 50 years of age carry germline *BRCA1* mutations [5]. Furthermore, 20% of TNBC patients have mutations of other genes involved in DNA repair pathways [6,7,8]. These deregulated pathways can be targeted by poly (ADP-ribose) polymerase inhibitors (PARPi) [9]. The U.S. Food and Drug Administration (FDA) approved PARPis, such as Olaparib, Niraparib, Rucaparib and Talazoparib, are being actively examined as monotherapy or in combination with other agents in breast cancer [10,11]. The majority of PARPi clinical trials (e.g., FDA approved Talazoparb) were designed to enroll patients harboring *BRCA1/2* germline mutations [12,13]. These trials revealed that PARPi therapy could benefit patients with germline *BRCA* mutations with a 12.9–62.6% response rate as monotherapy and 22–75% when combined with chemotherapy [12,13,14,15,16,17]. However, new biomarkers and new combination therapies of PARPi with other targeted agents need to be identified to benefit the patients with *BRCA*-proficient but DNA repair compromised pathways.

One biomarker that mediates replicative stress in cancer is cyclin E. Full-length cyclin E is a key cell cycle regulator with multiple functions including the activation of CDK2 and the maintenance of genome integrity. In normal cells, cyclin E is localized to the nucleus. In tumor cells, however, cyclin E accumulates in the cytoplasm and is of low molecular weight forms [18]. These truncated isoforms are collectively termed low molecular weight cyclin E (LMWE) [18]. In both normal and tumor cells and tissues, full length cyclin E is expressed at approximately 50 kDa, as determined by Western blot and mass spectrometry analyses [19]. In tumor cells and tissues, five tumor-specific LMWE isoforms with molecular weights ranging from 45 to 33 kDa have been identified [19,20]. Digestion of cyclin E with neutrophil elastase (NE) recapitulates the characteristic LMWE pattern seen in cancer cells [19,21]. In addition, at least two other proteases, calcium-dependent calpain [22] and calpain-2 [23], have been linked to the generation of LMWE in tumor cells. Clinically, overexpression of LMWE has been reported in multiple malignancies including breast [18], colorectal cancer [24] and lung cancer [25], reviewed in [26]. In breast cancer, we have reported that LMWE expression can be used to stratify patients with the highest likelihood of recurrence, across all subtypes [27,28,29,30]. Further, high LMWE expression is predictive of a poor pathological complete response to neoadjuvant chemotherapy [27].

*BRCA1/2* can also serve as biomarkers since reduced expression of *BRCA1/2*, due to mutations or epigenetic inactivation, leads to impaired mammary gland differentiation and increased risk of breast cancer development [31]. Furthermore, *BRCA1/2* germline mutations are associated with prolonged survival in TNBC, because the tumors with these mutations are more sensitive to therapies, such as platinum, alkylating agents, anthracyclines or PAPP inhibitors [32].

PARP stabilizes reversed forks during DNA replication and prevents fork collapse [33,34]. Furthermore, inhibition of PARP enhances stress on the replication forks [35,36]. It is therefore plausible that cancer cells with high replicative stress, even in the absence of *BRCA1/2* mutations, may also be sensitive to PARPi. The overexpressed LMWE forms can also induce replicative stress [37,38,39]. Therefore, we hypothesize that breast cancers with either high levels of LMWE or harboring *BRCA1/2* mutations are more sensitive to PARPi than those breast cancers with low levels of cyclin E and wildtype *BRCA1/2*.

Cells that undergo replicative stress depend on Wee1 kinase, a key regulator of G2 to M check point, by negatively regulating entry into mitosis through inactivation of CDK1, arresting cells in G2/M phase of the cell cycle and allowing for DNA repair [40,41,42,43]. Furthermore, cancer cells with a deregulated G1 to S transition rely on Wee 1 kinase activity for repair of damaged DNA while arrested in G2/M [44,45]. Therefore, inhibition of Wee1 kinase activity abrogates the G2/M arrest propelling the cancer cells with a deregulated G1 to transition into premature mitosis, resulting in cell death through mitotic catastrophe or apoptosis [42,43,46,47]. High levels of full length or LMW cyclin E result in replicative stress and abrogation of the G1 to S check point, requiring cells to repair the damaged DNA at the G2/M checkpoint. We recently reported that TNBC cells with high cyclin E expression are more sensitive to Wee1 kinase inhibition than those with low levels of cyclin E [39]. The combination of PARP and Wee1 kinase inhibition has been investigated in non-small cell lung, ovarian and pancreatic cancers, as well leukemia [35,48,49,50]. However, the efficacy of this combination has not been assessed in TNBC cells expressing either high levels of LMWE or harboring *BRCA* mutations.

Here, we report that breast cancers with mutations in DNA repair genes exhibit higher levels of cyclin E than those without such mutations. Additionally, breast cancers with alterations of cyclin E have a higher prevalence of germline *BRCA1/2* mutations than other patients. Most importantly, among patients with *BRCA1/2* mutations, high cyclin E levels are associated with worse outcomes. Lastly, the efficacy and mechanisms of the combination of PARP and Wee1 kinase inhibition are assessed in vitro and in vivo in TNBC cells and xenograft models with either *BRCA1* mutations or high levels of cyclin E.

## 2. Results

### 2.1. High Cyclin E RNA and Protein Levels Predict Poor Outcome in Breast Cancer Patients with BRCA1/2 Mutations

**Figure 1:**
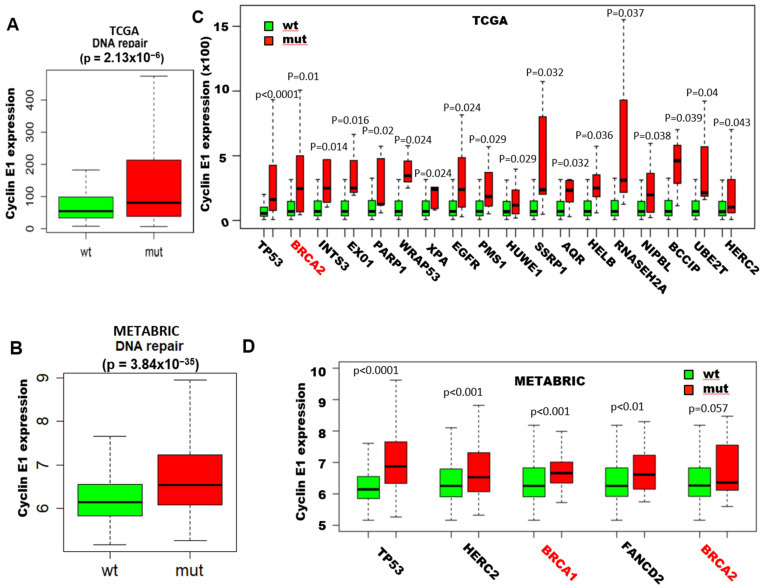

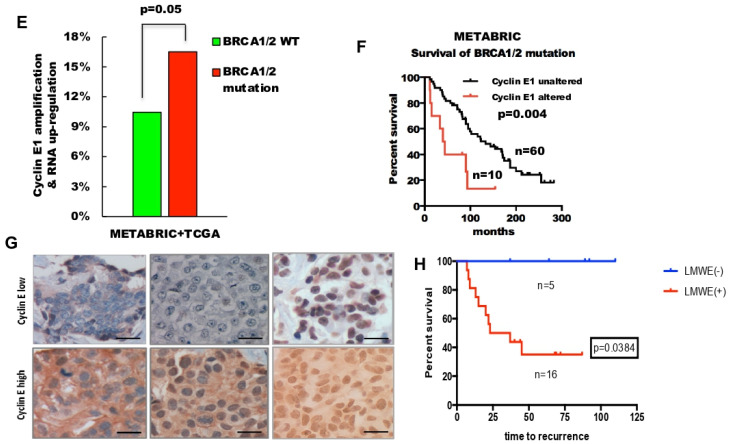
Cyclin E predicts poor outcome in breast cancer patients with *BRCA1/2* mutations. (**A**,**B**) Cyclin E expression was compared between breast cancer patient tumor samples from (**A**) The Cancer Genome Atlas (TCGA) and (**B**) Molecular Taxonomy of Breast Cancer International Consortium (METABRIC) databases with and without mutations in the DNA repair pathway (GO_DNA_REPAIR M12606). (**C**,**D**) Box plots of individual genes in the DNA repair pathway with and without mutations as a function of cyclin E expression in breast cancer patient cohorts from (**C**) TCGA (**D**) and METABRIC databases. (**E**) Percentage of breast tumors with cyclin E amplification and/or mRNA upregulation in the samples with or without *BRCA1/2* mutation from the combined TCGA and METABRIC databases. (**F**) Kaplan–Meier survival plot of cyclin E alterations (mRNA and DNA) of the patient cohorts with *BRCA1/2* mutations from the METABRIC database. (**G**) Representative cyclin E immunohistochemistry (IHC) of *BRCA1* mutated breast tumor tissues from patient samples with low (top) or high (bottom) cyclin E protein levels (scale bar, 20 μm). (**H**) Kaplan–Meier survival plot of cyclin E based on IHC analysis results (high/low) from 21 breast samples with *BRCA1* mutation.

Since high levels of cyclin E have been associated with increased genomic instability, we initially examined whether overexpression of cyclin E is also associated with mutations in DNA repair genes in breast cancer patients. To this end, we interrogated the mutational status of 544 known genes of the DNA repair pathway (GO_DNA_REPAIR M12606) among all breast cancer patients from The Cancer Genome Atlas (TCGA) (Figure 1A) and the Molecular Taxonomy of Breast Cancer International Consortium (METABRIC) databases (Figure 1B), as a function of cyclin E gene expression. Compared to tumor samples with no mutations, the samples harboring mutations in any of these 544 DNA repair genes have significantly higher expression of cyclin E. Specifically, among these 544 genes, there are 18 genes in the TCGA data base and 5 genes in the METABRIC data base whose mutation status were significantly associated with high cyclin E expression in patient samples examined (Figure 1C,D). These results revealed that amongst all the genes examined, *BRCA1/2* mutation is associated with the highest rank to cyclin E1 expression in both the TCGA (Figure 1 C) and METABRIC (Figure 1D) databases. Specifically, *BRCA2* mutation is the second and fifth highly associated mutation in the TCGA and METABRIC databases, respectively, while *BRCA1* mutation is the third highly associated mutation in the TCGA data base correlated with high cyclin E levels. TP53 is the only other mutated gene that was also highly associated with cyclin E expression in both databases. However, since mutations in *BRCA1/2* and p53 are closely correlated to each other [51,52,53], and since *BRCA1/2* mutation carriers are highly sensitive to PARP inhibitors, we selected *BRCA1/2* mutations for further analysis. Our analysis revealed that, in fact, mutations in *BRCA1* and *BRCA2* genes were amongst these mutated genes associated with high cyclin E expression levels (Figure 1E, Supplementary Materials Figure S1). Next, we asked if the addition of cyclin E as a biomarker would help differentiate the outcome of patients whose tumors harbor mutations in either *BRCA1* or *BRCA2*. The results revealed that those *BRCA1/2* mutation carriers had a worse outcome if their tumors also harbored cyclin E alterations (Figure 1F). We next examined the correlation between cyclin E protein alteration and *BRCA1* by immunohistochemical staining of cyclin E in 21 breast cancer samples from patients who have germline mutations in *BRCA1* treated at the MD Anderson Cancer Center (Figure 1G,H). The images on the top panels of Fig 1G depict the normal expression of cyclin E, which is either very low expression or just the nuclear expression of cyclin E. The images on the bottom panels of Fig 1G depict the tumor specific expression of cyclin E which shows its cytoplasmic subcellular localization. Results revealed that amongst these *BRCA1* mutation carriers, those who had overexpression of the cytoplasmic, tumor specific forms of cyclin E (i.e., LMWE forms) by immunohistochemistry (IHC) (Figure 1G) had significantly shorter time to recurrence compared to those patients whose tumors had low or just nuclear expression of cyclin E (Figure 1G,H). Together, these results suggest that cyclin E levels are associated with the DNA repair pathway mutations. Moreover, the *BRCA1/2* breast cancer mutation carriers with elevated cyclin E levels have a higher recurrence rate than those with low-cyclin E levels.

### 2.2. Triple-Negative Breast Cancers (TNBC) with Low-Molecular-Weight Cyclin E (LMWE) Are Sensitive to PARP Inhibition

**Figure 2:**
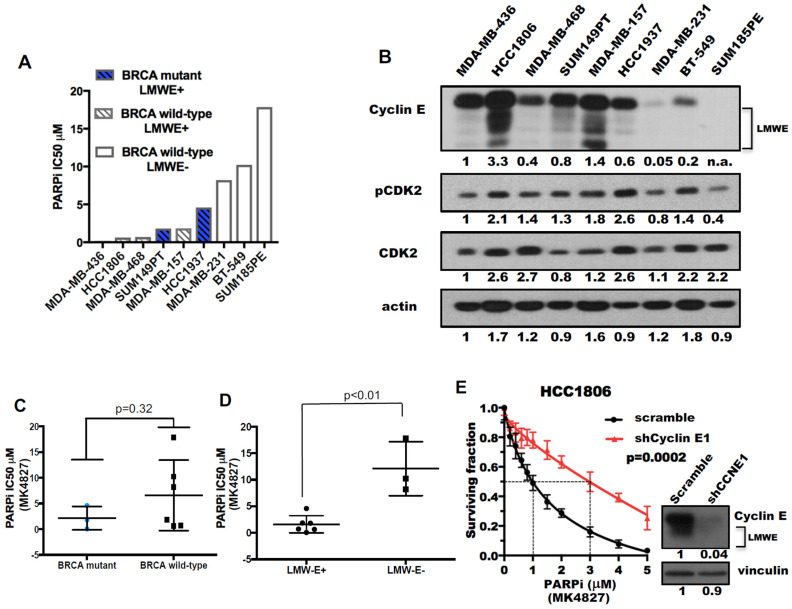

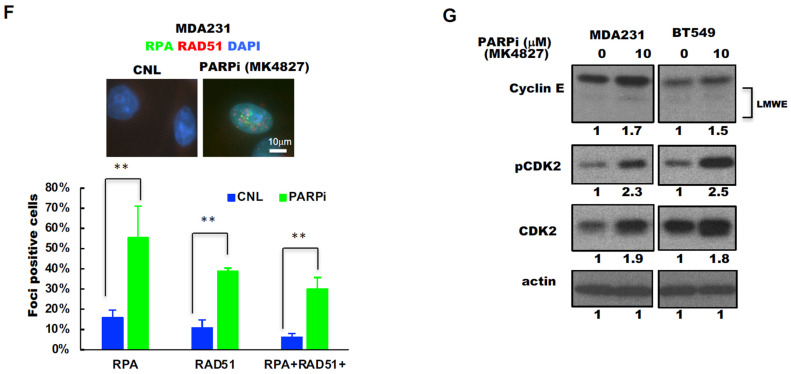
Triple-negative breast cancers (TNBCs) with low molecular weight cyclin E (LMWE) expression are sensitive to poly (ADP-ribose) polymerase (PARP) inhibition. (**A**) The IC_50_ of the PARP inhibitor (MK-4827) in 9 TNBC cells as a function of *BRCA1/2* mutation status and LMWE expression. (**B**) Western blots of 9 TNBC cells with indicated antibodies. Actin was used as loading control. (**C**,**D**) the comparison of IC_50_ of PARP inhibitor in 9 TNBC cells (from **A**) as a function of (**C**) *BRCA1/2* mutational status or (**D**) LMWE status. A two-tailed unpaired *t*-test was used to compare the two groups in each panel. (**E**) HCC1806-shRNA cyclin E cells were subjected to high through put survival analysis with increasing concentrations of PARPi (MK-4827) for 48 h as shown in Figure S2A and were immunoblotted (inset) with the indicated antibodies. A two-tailed paired *t*-test was used to compare two groups. (**F**) Representative images (top) and quantification of foci-positive cell (>5 foci per cell, bottom) population of RPA and RAD51 in MDA231 cells treated with 5 μM MK-4827 (PARPi) for 48 h. RPA+RAD51 indicates cells with both RPA and RAD51 foci. Scale bar, 10 μm. *n* = 3. A two-tailed unpaired *t*-test was used to compare two groups. Error bars represent standard error of the mean. **, *p* < 0.01. (**G**) MDA231 and BT549 cells were treated with 10 μM MK-4827 (PARPi) for 48 h and subjected to Western blots with the indicated antibodies. CNL, control. Vinculin was used as loading control. Densitometry was performed on all western blots and the relative expression of each band to its loading control is noted on the bottom of each panel for each antibody used. n.a. densitometry is not available because the western blot band cannot be detected.

We next set out to identify treatment strategies that could be effective in cells with either high cyclin E expression (i.e., high LMWE) or those harboring *BRCA1/2* mutations. Since cell lines with *BRCA1/2* mutations are likely to be sensitive to PARP inhibitors (PARPi), such as MK-4827, we compared the sensitivity of 9 TNBC cell lines with different *BRCA1/2* mutations status and expression levels of LMWE to PARP inhibition using a high-throughput-survival assay (HTSA) (Figure 2A,B, Figure S2A). Results revealed that LMWE expression was a better biomarker of response to PARPi (MK-4827) than *BRCA1/2* status (Figure 2C,D). Specifically, TNBC cell lines with high LMWE expression were more sensitive to PARPi (MK-4827), with a 6-fold lower IC50, than the low LMWE expressing cells (Figure 2D). Knockdown of cyclin E in HCC1806 decreased the sensitivity of this cell line to PARPi (MK-4827) by 3-fold (from an IC50 of 1 μM to 3 μM) as compared to scrambled control cells (Figure 2E, Figure S2A). Conversely, induction of LMWE in the 76NE6 human mammary epithelial cell line increased sensitivity to PARPi (MK-4827) compared to vector control cells (Figure S2B–D). We next examined changes in RPA foci, a marker of DNA replication stress, and RAD51 foci, a marker of DNA repair in the LMWE-low MDA231 cells treated with or without 5 μM MK-4827 (PARPi) for 48 h (Figure 2F, Figure S2E). PARP inhibition increased the RPA-foci positive cells by 3-fold (from 16% in vehicle treated cells to 55% in PARPi (MK-4827) treated cells) and the RAD51-foci positive cells by 4-fold (from 11% in vehicle treated cells to 40% in MK-4827 treated cells). The PARPi (MK-4827) mediated increases in Rad51 and RPA foci were associated with an increase in p-CDK2 expression suggesting activation of CDK2. These results also suggest that since treatment with PARP inhibition can increase replicative stress (i.e., induction of RPA and RAD51 foci), combination therapy of PARPi (MK-4827) with agents targeting replicative stress, such as Wee1 kinase inhibitor, could be synergistic in TNBC cells that harbor either *BRCA1/2* mutations or cyclin E overexpression.

### 2.3. The Combination of PARP Inhibitor (PARPi) and Wee1 Kinase Inhibitor (Wee1i) Is Synergistic by Inducing Apoptosis in TNBC Cells

**Figure 3:**
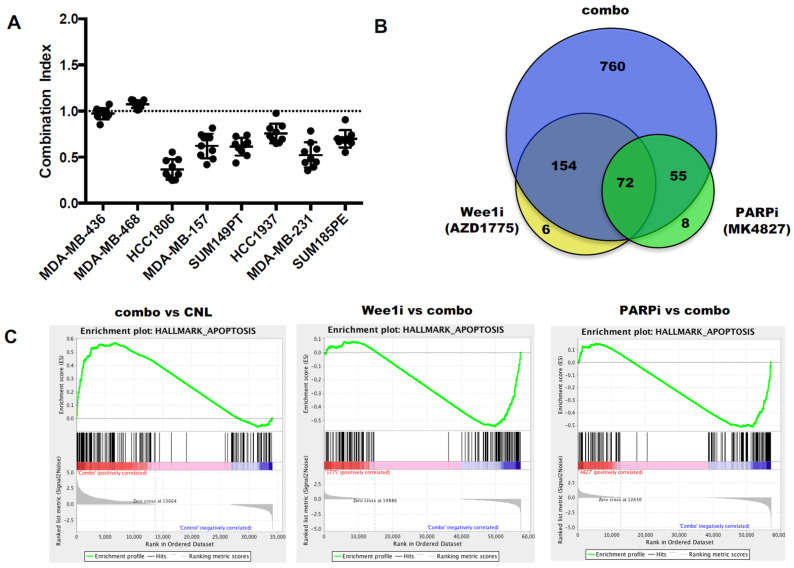

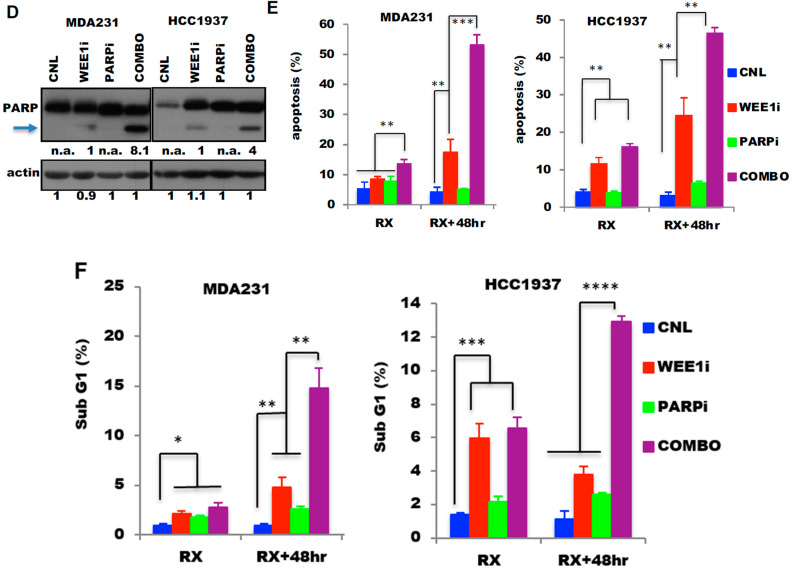
The combination of PARP inhibitor (PARPi) and Wee1 kinase inhibitor (Wee1i) is synergistic by inducing apoptosis in TNBC cells. (**A**) Combination index (CI) of the 48-h combination treatment of PARPi (MK-4827) and Wee1i (AZD-1775) in TNBC cells. CI = 1.0 indicates an additive effect (dotted line), CI > 1.0 indicates antagonism and CI < 1.0 indicates synergism. (**B**) Venn diagrams of the RNA sequencing results of MDA231 cells treated for 48 h with AZD-1775 (Wee1i), MK-4827 (PARPi) or both drugs (combo) and changed to the drug-free media for 48 h at which point they were subjected to RNA sequencing. The results have been normalized by the vehicle control cells. (**C**) The apoptosis gene set enrichment by GSEA analysis of the RNA sequencing results. Left panel comparing combo versus CNL (vehicle) treatment shows that apoptosis is enriched in the combo treated arm; Middle panel comparing Wee1i versus combo treatment shows that apoptosis enriched in combo; Right panel comparing PARPi versus combo treatment shows that apoptosis is enriched in combo. (**D**–**F**) MDA231 (right) and HCC1937 (left) cells were treated for 48 h with: CNL, vehicle treated control; Wee1i, AZD-1775 (2.5 μM for MDA231 and 0.15 μM for HCC1937); PARPi, (5 μM MK-4827 (PARPi) for both MDA231 and HCC1937); and COMBO, the combination treatment of Wee1i (AZD-1775) and PARPi (MK-4827) as the same dosage of monotherapy-treatment for each cell line and subjected to (**D**) Western blot analysis with PARP and Actin; Arrow shows the cleaved PARP and the densitometry of PARP is the cleaved band. Densitometry was performed on all Western blots and the relative expression of each band to its loading control is noted on the bottom of each panel for each antibody used. n.a. densitometry is not available because the Western blot band cannot be detected (**E**) Annexin V assay following 48 h of treatment (RX-left panels) or 48 h of treatment followed by 48 h in drug free medium (RX + 48 h right panels) and (**F**) flow cytometry analysis and sub-G1 quantitation in the RX and RX + 48 h treated cells (as in (**E**)). A two-tailed unpaired *t*-test was used to compare two groups. Error bars represent standard error of the mean. *, *p* < 0.05; **, *p* < 0.01; ***, *p* < 0.001; ****, *p* < 0.0001.

To investigate the efficacy of the combination treatment of the PARPi (MK-4827) and a Wee1i (AZD-1775) in TNBC cells, we subjected 8 different TNBC cell lines to treatment with single agents or a combination of PARPi (MK-4827) and Wee1i (AZD-1775) using a long-term (12 days) high throughput survival assay (HTSA schematically depicted in Figure S3A) [54]. The majority (6 out of 8) of the TNBC cell lines treated with a combination of PARPi (MK-4827) and Wee1i (AZD-1775) showed a synergistic combination index (CI) of 0.2–0.9 as calculated by the CalcuSyn software (Figure 3A).

To understand the molecular regulation of the synergistic combination treatment, we performed RNA sequencing analysis in MDA231 cells (that showed synergism between MK-4827 and AZD-1775) treated with (i) vehicle, (ii) 5 μM MK-4827 (PARPi), (iii) 2.5 μM AZD-1775 (Wee1i) and (iv) a combination of PARPi (MK-4827) and Wee1i (AZD-1775). Compared to the vehicle control, the combination treatment resulted in 1041 differentially expressed genes (DEGs) while the single PARPi (MK-4827) and Wee1i (AZD-1775) treatments resulted in 135 and 232 DEGs, respectively (Figure 3B). Among all DEGs, 72 were shared by all three treatment regimens; 154 DEGs shared between only the combination and Wee1i (AZD-1775) treatment; and 55 DEGs were shared between only the combination and PARPi (MK-4827) treatment. Gene set enrichment analysis (GSEA) revealed that the combination treatment enriched the genes involved in apoptosis compared to the other arms (Figure 3C), although both the Wee1i and PARPi treatments were able to enrich apoptosis-associated genes compared to the control arm (Figure S3B). These results suggested that the combination of the two drugs could accelerate the apoptotic process in MDA231 cells. To validate the RNA sequencing analysis, we subjected two TNBC cell lines (MDA231 and HCC1937) that showed a synergistic CI with the combination treatment (i.e., CI 0.2–0.5 Figure 3A) to (1) Western blot analysis for cleaved PARP (Figure 3D), (2) Annexin/PI apoptotic assay (Figure 3E) and (3) cell cycle analysis to examine the sub-G1 population (Figure 3F). Results revealed that the combination treatment increases cleaved PARP levels in both cell lines. Apoptosis assays and cell cycle analysis revealed that the apoptotic and sub-G1 populations of both cell lines were significantly increased compared to other groups, especially under conditions when cells were released from the combination treatment for 48 h (RX + 48 h), allowing them to accumulate DNA damage that may not be repaired, thus inducing apoptosis. This latter hypothesis was directly tested in subsequent studies.

### 2.4. Combination of PARP and Wee1 Kinase Inhibition Increases DNA Replicative Stress and Polyploidy

**Figure 4:**
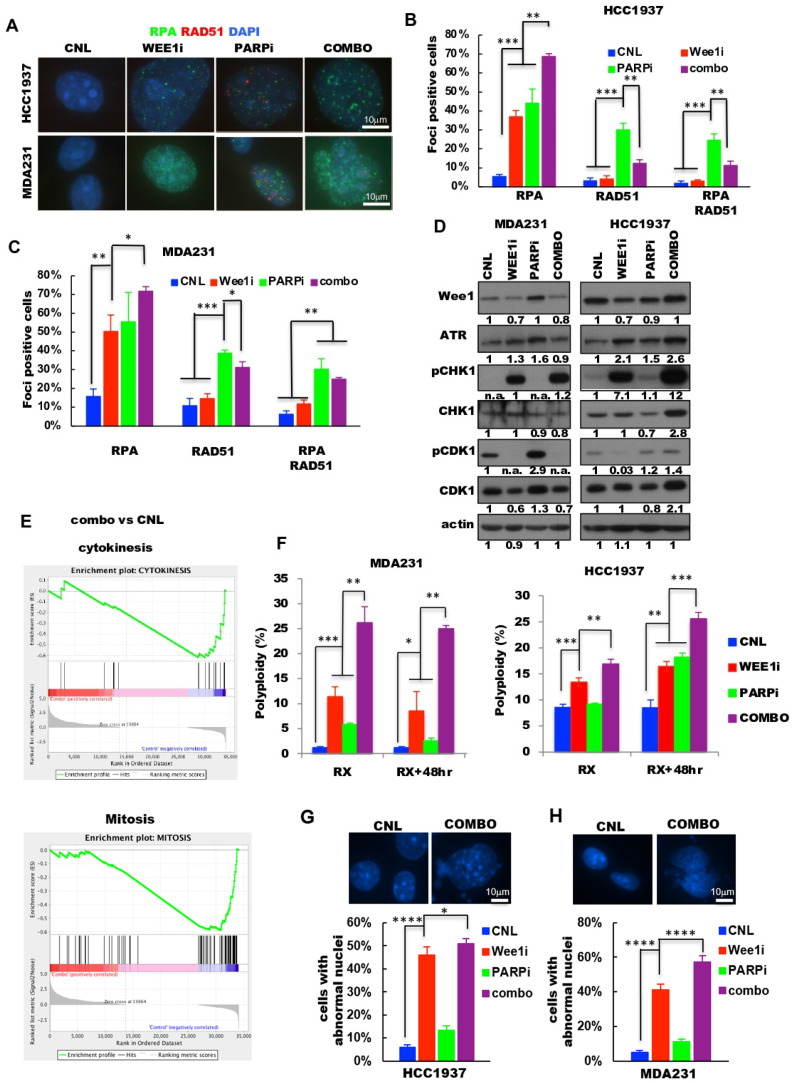
PARP inhibition sensitizes cells to Wee1 inhibitor by increasing DNA replication stress. (**A**) Representative images of RPA (green) and RAD51 (red) foci in HCC1937 (top) and MDA231 (bottom) cells treated with CNL, vehicle control; Wee1i, AZD-1775 (2.5 μM for MDA231 and 0.15 μM for HCC1937); PARPi, 5 μM MK-4827 for both cell lines; COMBO, the combination treatment of Wee1i (AZD-1775) and PARPi (MK-4827) as the same dosage of monotherapy-treatment for 48-h. Scale bar, 10 μm. (**B**,**C**) Quantification of foci-positive cells from A (>5 foci per cell, bottom) in (**B**) HCC1937 and (**C**) MDA231 cells. RPA+RAD51 was quantitated for cells with both RPA and RAD51 foci. (**D**) Western blot analysis with the indicated antibodies in MDA231 (right) and HCC1937 (left) cells with the treatment arms as in (**A**). Actin served as loading control. Densitometry was performed on all Western blots and the relative expression of each band to its loading control is noted on the bottom of each panel for each antibody used. n.a. densitometry is not available because the Western blot band cannot be detected. (**E**) Gene set enrichment analysis (GSEA) for cytokinesis (top) and mitosis (bottom) gene sets using the RNA sequencing results comparing combo versus CNL. The analyses indicated that both cytokinesis and mitosis gene sets enriched in CNL as compared to combo. (**F**) Percent polyploid cells (as determined from flow cytometry analysis) of cells with the same treatment arms as **A** following 48 h of treatment (RX-left panels) or 48 h of treatment followed by 48 h in drug free medium (RX + 48 h right panels). (**G**,**H**) Representative images (top) of normal (CNL) and abnormal (COMBO) nuclei and their quantification (bottom) of the frequency of abnormal nuclei in (**G**) HCC1937 and (**H**) MDA231 cells with the treatment arms as in A. Scale bar, 10 μm. A two-tailed unpaired *t*-test was used to compare two groups. Error bars represent standard error of the mean. *, *p* < 0.05; **, *p* < 0.01; ***, *p* < 0.001; ****, *p* < 0.0001. All experiments were repeated 3 times.

GSEA analysis from the RNA sequencing of the combination treatment of PARP and Wee1 kinase inhibitors revealed that compared to either vehicle or single drug treatments, the combination treatment resulted in the down-regulation of genes regulating DNA replication (Figure S3C). These results raised the testable hypothesis that the combination treatment may induce more DNA replicative stress than either drug alone. To test this hypothesis, RPA (a marker of DNA replication stress) and Rad51 (a marker of DNA repair) foci were enumerated in MDA231 and HCC1937 cells treated with the four treatment arms (vehicle, each of the two drugs individually and in combination) (Figure 4A–C, Figure S4). Results revealed that the RPA-foci positive cells were significantly higher in the Wee1i or PARPi treated cells than the control arm in both cell lines. The combination treatment further increased the RPA-foci positive cells to 69% in HCC1937 cells (Figure 4B) and 72% in the MDA231 cells (Figure 4C), suggesting that the majority of cells underwent DNA replicative stress upon treatment with the combination of PARPi (MK-4827) and Wee1i (AZD-1775). RAD51 foci, a measure of DNA repair, was most pronounced in cells treated with the PARP inhibitor. Western blot analysis revealed that several of the replicative stress mediators, which regulate traverse through mitosis, were also modulated by the combination treatment such as phosphorylated CHK1, ATR and Wee1 (Figure 4D), suggesting that inhibition of PARP and/or Wee1 kinase may themselves alter cell cycle progression. Cell cycle analysis revealed that compared to the vehicle treatment controls, treatment of cells with either individual drugs or in combination increased the percent of cells in S and G2/M phases of the cell cycle (Figure S5A).

GSEA analysis of the cytokinesis and mitosis gene sets revealed that these pathways were downregulated in cells treated with combination of PARPi and Wee1i, as compared to control cells or individual drug treatment (Figure 4E, Figure S5B–D), raising the testable hypothesis that when cells are treated with both PARPi and Wee1i, they are not likely to complete their cell division, resulting in polypoid cells with abnormal nuclei. Flow cytometric analysis showed that the combination treatment of cells significantly increases the polyploid population compared to vehicle or single treatment arms even following 48-h removal of the drugs (Figure 4F). Consistently, quantitation of multinucleation and micronucleation (i.e., abnormal nuclei) in all treatment arms revealed that 51% and 57% of HCC1937 and MDA231 cells, respectively, treated with the combination harbored abnormal nuclei as compared to 5–6% of the untreated controls (Figure 4G,H, Figure S5E). Collectively, these results suggest that treatment of TNBC cells with combination of PARP and Wee1 kinase inhibitors can accelerate replicative stress and severely deregulate the cell cycle progression of TNBC cells resulting in a high degree of polyploid cells with abnormal nuclei.

### 2.5. PARP and Wee1 Kinase Inhibition Deregulates DNA Repair and Increases DNA Damage

**Figure 5:**
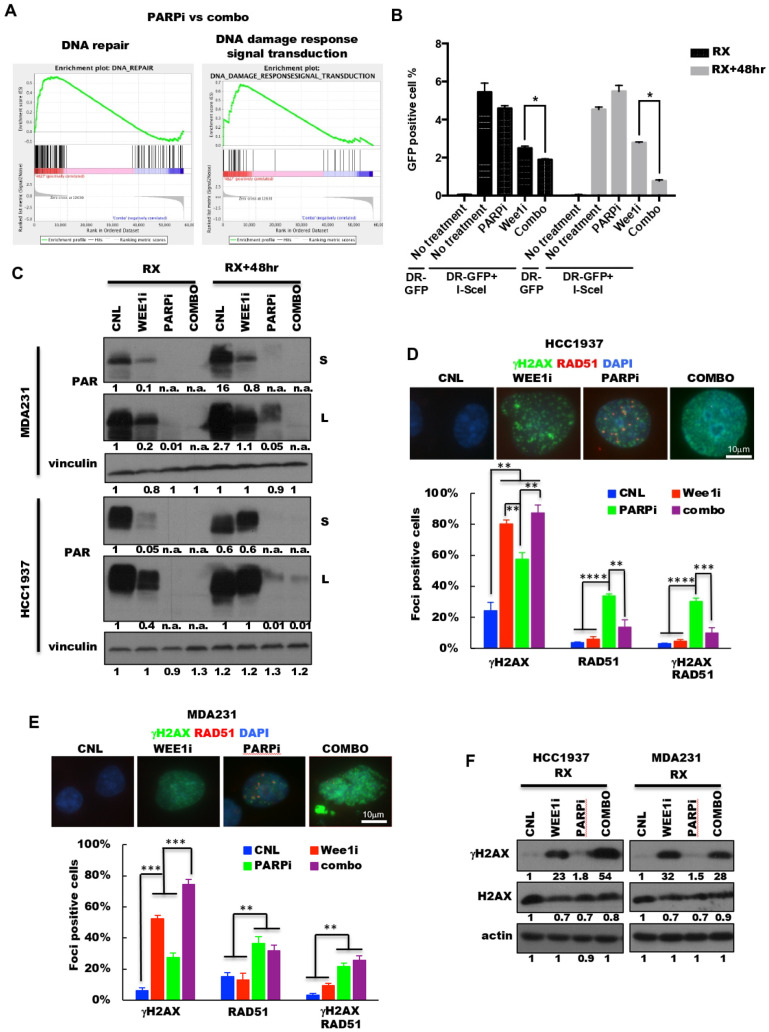
Wee1 inhibition sensitizes cells to PARP inhibitor by inhibiting DNA repair. (**A**) Gene set enrichment analysis (GSEA) for DNA-repair (left) and the DNA-response-signal-transduction (right) gene sets using the RNA sequencing results comparing PARPi versus combo. The analyses indicated that both DNA repair and DNA damage response signal transduction gene sets are enriched in PARPi treated cells. (**B**) DR-GFP homologous recombination repair assay: MDA231 cells were transfected with DR-GFP reporter assay and 24 h post transfection, cells were treated with CNL, vehicle control; Wee1i, AZD-1775 (2.5 μM); PARPi, 5 μM MK-4827 or COMBO, the combination treatment of Wee1i (AZD-1775) and PARPi (MK-4827) 48-h (RX) or changed to the drug-free media for 48 h (RX + 48 h) followed by FACS analysis for GFP positive cells. Values are normalized by control group. Error bars represent mean ± SD (*n* > 3 independent experiments). (**C**) Western blot analysis with indicated antibody in MDA231 (top) and HCC1937 (bottom) cells with the treatments as (**B**). Vinculin was used as loading control. Densitometry was performed on all Western blots and the relative expression of each band to its loading control is noted on the bottom of each panel for each antibody used. n.a. densitometry is not available because the Western blot band cannot be detected. (**D**,**E**) Representative images of γH2AX (green) and RAD51 (red) foci (top) and quantification of foci-positive cell (>5 foci per cell, bottom) population in (**D**) HCC1937 and (**E**) MDA231 cells treated with the 48-h treatment as (**B**). Scale bar, 10 μm. RPA+RAD51 shows cells with both RPA and RAD51 foci. *n* = 3. (**F**) Western blot analysis with indicated antibodies in HCC1937 (right) and MDA231 (left) cells with the same treatments as in (**B**). Actin was used as loading control. Densitometry was performed on all Western blots and the relative expression of each band to its loading control is noted on the bottom of each panel for each antibody used. A two-tailed unpaired *t*-test was used to compare two groups. Error bars represent standard error of the mean. *, *p* < 0.05; **, *p* < 0.01; ***, *p* < 0.001; ****, *p* < 0.0001.

We next assessed if treatment of cells with both PARP and Wee1 inhibitors would downregulate DNA repair capacity of the treated cells. GSEA analysis of the RNA sequencing of MDA231 cells revealed that treatment of cells with combination PARPi (MK-4827) and Wee1i (AZD-1775) decreased both the DNA repair and DNA damage response signal transduction gene sets, as compared to PARPi or Wee1 kinase treatment alone (Figure 5A, Figure S6). To directly examine if the combination treatment induced inhibition of DNA repair, we measured homologous recombination (HR) repair using the DR-GFP/I-Scel HR assay in MDA231 cells (Figure 5B). The results revealed that cells treated with the combination of both drugs had the lowest percentage of HR repair at the 48-h treatment (RX) or after the drug removal (RX + 48 h). To explore the single strand damage repair pathway, we examined the expression of Poly ADP-ribose (PAR) levels by Western blot analysis. PAR protein is rapidly synthesized by PAR polymerases (PARPs) upon activation of DNA single and double strand breaks [55]. Results revealed that while treatment of MDA231 and HCC1937 cells with PARPi (MK-4827) reduced the PAR levels in both cell lines, those cells treated with both PARPi (MK-4827) and Wee1i (AZD-1775) resulted in more significant inhibition of PAR levels (Figure 5C), suggesting that Wee1 kinase inhibition facilitated the ability of PARP inhibitor to suppress DNA single and double strand damage repair in both cell lines. To further test this hypothesis, we asked if combination of PARP and Wee1 kinase inhibition can induce DNA replication stress in both cell lines, as a result of accumulated and unrepaired DNA damage. Gamma-H2AX (γH2AX) foci were enumerated in HCC1937 and MDA231 cells in the vehicle control (CNL), the AZD-1775 treatment alone (Wee1i), the MK-4827 treatment alone (PARPi) and the combination treatment of both agents (combo) (Figure 5D,E, Figure S7). The results revealed that the γH2AX-foci positive cells of the combination treatment had increased to 87% in HCC1937 cells and 74% in MDA231 cells, which are significantly higher than the monotherapy-treatment arms. Additionally, the γH2AX protein expression in the combo treated arms were significantly higher than the control or PARP treated arms in both cell lines (Figure 5F). Collectively, these results suggest that the combination treatment with PARPi (MK-4827) and Wee1i (AZD-1775) suppresses multiple DNA repair pathways causing extensive DNA damage, which may lead to cell death.

### 2.6. The Combination of PARP and Wee1 Kinase Inhibition Inhibited Tumor Growth and Improved Survival In Vivo

**Figure 6:**
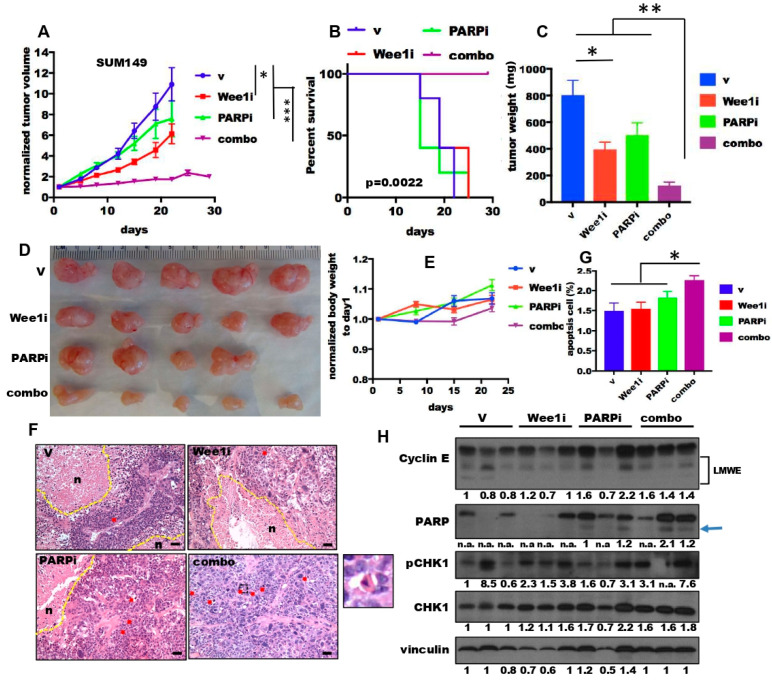
Combination treatment of Wee1 kinase and PARP inhibitors inhibit tumor growth in TNBC xenograft models (**A**–**C**) SUM149 cells were injected into the fat pads of immunocompromised mice and allowed to grow to approximately 150 mm^3^; xenografts were treated with either vehicle (V), 50 mg/kg AZD-1775 (Wee1i), 50 mg/kg MK-4827 (PARPi) and both drugs (combo) 5 times weekly for up to 4 weeks or until tumors reached maximum IACUC allowable tumor volume (see schema in Figure S8A). (**A**) Tumor volume measurement and (**B**) Kaplan–Meier survival analysis of mice treated with the indicated drug treatment arms. (**C**,**D**) Tumors from mice treated with each of the treatment arms were extracted and weighed and (**C**) tumor weight at the end of the treatment plotted. (**E**) Mouse weights in response to treatment. (**F**) Representative hematoxylin and eosin staining of the treated tumors at the end of treatment depicting necrosis (n) and yellow dotted lines or apoptotic cells which are highlighted by red arrows. The inset is a representative apoptotic cell at high magnification. Scale bar, 20 μm. (**G**) Quantification of the apoptotic cells from panel F. (**H**) Tumors from mice from each of the treatment arms were extracted and subjected to Western blot analysis of cyclin E, PARP, pCHK1 and CHK2 and actin. Arrow shows the cleaved PARP and the densitometry of PARP is the cleaved band. Densitometry was performed on all Western blots and the relative expression of each band to its loading control is noted on the bottom of each panel for each antibody used. n.a. densitometry is not available because the Western blot band cannot be detected. A two-tailed unpaired *t*-test was used to compare two groups. *n* = 4–19. Error bars represent standard error of the mean. The log-rank (Mantel–Cox) test was used in survival experiments. *, *p* < 0.05; **, *p* < 0.01; ***, *p* < 0.001.

We next examined the in vivo efficacy of combination treatment of PARPi (MK-4827) and Wee1i (AZD-1775) in two TNBC xenografts: SUM149 (Figure 6) and HCC1806 (Figure S8). SUM149 tumors harbor *BRCA1* mutations and allelic loss [56], while HCC1806 have a wild type *BRCA1* [57], but overexpress LMWE (see Figure 2A,B). Mice harboring the orthotopic TNBC xenografts were randomized into 4 treatment arms: (1) Vehicle (v), (2) 50 mg/kg AZD-1775 alone (Wee1i), (3) 50 mg/kg MK-4827 alone (PARPi), (4) 50 mg/kg AZD-1775 and 50 mg/kg MK-4827 (combo). Mice received daily treatments 5 days per each 7-day cycle (Figure S8A). In both xenograft models, the combination treatment significantly inhibited tumor growth during the 3 cycles of treatment (Figure 6A and Figure S8B) resulting in prolonged survival of the tumor bearing mice (Figure 6B), as compared to single treatment arms. Moreover, the combination treatment resulted in 85% decrease in tumor volume at the end of the 3 cycles of treatment versus 50% in the single treatment arms, as compared to vehicle treated controls (Figure 6C,D). Notably, the mouse body weights of all the treatment arms did not decrease during the course of treatment, suggesting limited toxicity of these treatment regimens (Figure 6E).

To investigate the cell death events, we examined the histology of the tumors with the treatments at the end of 3 cycles (Figure 6F). The tumors with the combination treatment exhibited much less necrosis than the tumors of other arms, possibly due to the smaller size of the tumors. However, the combination treated tumors had a significant increase in apoptosis, as indicated by the apoptotic bodies compared with the single treatment arms (Figure 6G). Consistently, the levels of cleaved PARP increased in the combination treated and the PARPi treated arms, as compared to the Wee1i and the vehicle treated arms (Figure 6H). Moreover, the levels of phospho-CHK1 also increased in the tumors from the combination treatment arm. Collectively, these studies suggest that combination treatment with a PARPi (MK-4827) and Wee 1i (AZD-1775) can synergistically inhibit tumor growth in vivo by inducing apoptosis, similar to the in vitro cell line data presented.

## 3. Discussion

In this report, we show that breast cancer patients whose tumors show high levels of cyclin E expression also have a higher prevalence of DNA repair gene (e.g., *BRCA1/2*) alterations compared to those patients without cyclin E overexpression. We also show that among breast cancer patients who have germline *BRCA1/2* mutations, if their tumors also show high cyclin E RNA or high levels of LMWE protein expression, they have worse clinical outcomes. The association between cyclin E gene amplification and *BRCA1/2* mutations have been examined previously and in studies with high grade serous ovarian cancer, such associations were found to be mutually exclusive [58,59]. However, when correlating cyclin E protein levels to *BRCA1/2* mutations, studies in both ovarian and breast cancer patients show a significant level of correlation [60,61,62]. While mechanisms of regulation between *BRCA1/2* and cyclin E remain unclear, a recent study suggested that BRCA1 mutation may stabilize cyclin E1 protein by decreasing phosphorylation T62 on cyclin E1 [62]. Phosphorylation T62 of full-length cyclin E1 [63], but not its LMWE forms, leads to its proteasome mediated degradation in the nucleus by FBWX7 [64].

Our in vitro and in vivo results reveal that combination therapy of these tumors with a Wee1 kinase inhibitor (AZD-1775), that targets cyclin E high tumors, and PARP inhibitor (MK-4827), that targets DNA repair gene mutations (e.g., *BRCA1/2*), provides a synergistic treatment option for these patients.

To understand how the combination treatment can differentially alter key signaling pathways, as compared to single agent treatment, at the molecular level, we performed RNA sequencing analysis on cells treated with either Wee1i (AZD-1775) or PARPi (MK-4827) monotherapy or combination therapy. Genes set enrichment analysis and subsequent validation studies revealed that several pathways were significantly altered only in the combination treated cells. For example, apoptosis and replicative stress pathways were increased while DNA repair pathways were downregulated, and cells also underwent cytokinesis failure in the combination treatment arms. In vivo, the combination of Wee1i (AZD-1775) and PARPi (MK-4827) resulted in synergistic tumor growth inhibition due to increased apoptosis which resulted in significant survival benefit in the two different human xenograft models we examined.

Our pre-clinical results also suggest that the combination treatment with Wee1 kinase (AZD-1775) and PARP (MK-4827) inhibitors are likely to be as effective in patients with only elevated cyclin E or BRCA mutations as in patients whose tumors harbor both alterations. For example, our in vitro combination index studies (Figure 3A) reveal that the efficacies of the combination treatment are similar between cell lines with both *BRCA* mutations and LMWE expression (SUM149PT and HCC1937) as well as cell lines with only LMWE expression (HCC1806, MDA-MB-157). In vivo, we also show that the synergism of the combination treatment strategy is similarly efficacious in HCC1806 xenograft model (*BRCA1/2* wild-type and LMWE expression) (Figure S8B) as compared to the SUM149T model (Figure 6A), harboring both *BRCA1* mutations and overexpression of cyclin E.

Collectively, these results suggest that targeting two main checkpoints in the cell cycle, G2/M (inhibited by a Wee 1 kinase inhibitor) and S phase (inhibited by a PARP inhibitor), results in synergistic cell death due to inhibition of both checkpoints (see Graphical Abstract for the model). Treatment of cells with Wee1 kinase inhibitor alone results in the activation of CDK1 that can result in unscheduled replication origin firing, depletion of dNTPs and replication factors [35,65,66] as well as leading to early mitotic entry and mitotic catastrophe [42], since cells are not allowed to repair DNA damage that occurs when CDK1/2 are inhibited (through phosphorylation) when Wee1 is not inhibited. Under these conditions the cell is more sensitive to DNA damage that is mediated through inhibition of PARP. As a result, following treatment of cells with both agents, the cells retain damaged DNA that cannot be repaired, undergo cytokinesis failure and mitotic catastrophe resulting in apoptosis. To this end, our results show that combination treatment of cells with the two agents leads to higher RPA and γH2AX foci (markers of DNA damage) and lower Rad51 foci (marker of DNA repair) as compared to single agent treatments (Figure 4A–C and Figure 5D,E). Consistently, the combination treated cells had the lowest percentage of homologous recombination repair, compared to single agent treatments (Figure 5B). Moreover, these cells also undergo cytokinesis failure resulting in increased polyploid population and accumulation of cells with abnormal nuclei (Figure 4E–H).

While studies in many cancer types, including ovarian [50], pancreatic cancer [67] and small cell lung cancers [68], have shown that combination treatment of pre-clinical models with both Wee1 kinase and PARP inhibitors are effective in inhibiting cell growth and/or decreasing tumor volume in vivo, these studies lack identification of biomarkers that could aid in patient selection for future clinical trials. Here, we show that TNBC cell lines that either have overexpression of the LMWE or mutations in *BRCA1* are very sensitive to the combination treatment with Wee1i (AZD-1775) and PARPi (MK-4827). We propose that by preselecting patients whose tumors have high LMWE and/or harbor mutations in *BRCA1*, only those cases with the highest replicative stress properties will be subjected to combination treatment and likely result in synergistic activity of the two agents. We also propose that these high LMWE or *BRCA1* mutant tumors are likely to be very sensitive to apoptotic effects of the combination treatment, allowing the use of lower doses (than the typical maximum tolerable dose) of each drug to reduce the toxicity to the patients.

## 4. Material and Methods

### 4.1. Clinical Samples

A retrospective collection of clinical samples (*n* = 21) was used in this study and was approved by the University of Texas, MD Anderson Cancer Center Institutional Review Board (protocol number LAB-00-222) and patients signed consent for participation. Female patients 18 years or older, with a diagnosis of clinical stage II-III breast cancer and a known *BRCA1* germline mutation, were consented and enrolled to this study.

### 4.2. Immunohistochemistry (IHC) and Scoring of Cyclin E

IHC was performed as described [29]. Briefly, sections (5 μm thick) of formalin-fixed paraffin-embedded (FFPE) samples were deparaffinized and rehydrated followed by incubation with 3% hydrogen peroxide and methanol. Antigen retrieval was carried out with 0.01 mmol/L citric acid–based buffer at pH 6.0 using a hot plate in a metal container for 15 min before immunostaining. After 1-h blocking for nonspecific staining, the sections were incubated with the primary antibody and followed by the secondary antibodies. Antibodies were detected using a VECTASTAIN Elite ABC Kit (PK6101 and PK6102; Vector Laboratories). Positive cells were visualized using the chromogenic substrate 3,3′-diaminobenzidine. Slides were counterstained with hematoxylin and mounted. Cyclin E expression (C-19, sc-198; Santa Cruz Biotechnology) and scoring were assigned as described previously [29]. Briefly, nuclear and cytoplasmic cyclin E staining scores were independently assigned according to their staining intensity (0 = no staining, 1 = weak staining, 2 = intermediate staining and 3 = strong staining). The nuclear and cytoplasmic scores were then combined to generate LMWE–low and LMWE–high phenotypes. LMWE–low phenotypes include any nuclear cyclin E score (0–3) and cytoplasmic cyclin E scores of 0 and 1. LMWE–high phenotypes include only those cases with cytoplasmic scores of 2 or 3, regardless of their nuclear scores.

### 4.3. Cell Lines

HEK-293T cells, used for lentiviral packaging, and the TNBC cell lines HCC1806, MDA-MB-436, MDA-MB-468, MDA-MB-157, BT-549 and MDA-MB-231 (MDA231), were obtained from the American Type Culture Collection and cultured as described previously [29,54]. The TNBC cell lines SUM149PT and SUM185PE were obtained from Asterand Bioscience and cultured in Ham F12 medium with 5% fetal bovine serum, 5 μg/mL insulin and 1 μg/mL hydrocortisone. All cells were free of mycoplasma contamination. Cell lines were identified and regularly (every 6 months) authenticated by karyotype and short tandem repeat analysis at the MD Anderson Cancer Center Characterized Cell Line Core facility.

### 4.4. High-Throughput Survival Assay (HTSA)

Cells were treated and their long-term growth inhibition examined in 96-well plates over an 11-day period, a method that allows analysis of cytotoxicity of one or more drugs in a wide range of adherent cell lines and provides results that are highly consistent with classic clonogenic assays, as described previously [54,69]. For single MK-4827 (PARP inhibitor) treatment, TNBC cells and HCC1806 with shRNA knockdown were seeded and 24 h post seeding were treated with increasing concentrations of the inhibitor. Following 48 h of treatment, PARPi (MK-4827) was removed from cells and fresh drug-free medium added. The treated cells were kept in culture for 8 days and the medium was refreshed every other day with drug free medium as depicted in the schema (Figure S2A), at which point viability was assessed by MTT as described [70]. For treatment of the 76NE6 cyclin E-inducible cells, cells were seeded overnight, cyclin E expression (EL and T1) was induced by treatment with 10 ng/mL doxycycline for 24 h or left uninduced as a control. Cells were then treated with various concentrations of PARPi (MK-4827) for 48 h. Upon removal of the drug, the treated cells were kept in culture for 7 days, and the medium, with or without doxycycline, was refreshed every other day as depicted in the schema (Figure S2B), at which point, viability was assessed by MTT as described [70]. For the combination treatment of PARPi (MK-4827) and Wee1i (AZD-1775), TNBC cells or 76NE6 cells were seeded in 96-well plates overnight. Cells were then treated with increasing concentrations of PARPi (MK-4827) and Wee1i (AZD-1775) for 48 h and the medium was refreshed every other day with drug free medium before MTT assay [70] as depicted (Figure S3A).

### 4.5. Immunoblot Assays

Immunoblots were performed as previously described [30]. Briefly, 50 μg of protein from each sample was subjected to electrophoresis and transferred to an Immobilon P membrane (Millipore, Burlington, MA, USA). Membranes were blocked for 0.5 h in BLOTTO (5% nonfat dry milk in TBS-T; 20 mmol/L Tris, 137 mmol/L NaCl, 0.25% Tween, pH 7.6) and incubated, first, with the primary antibodies and then, with the secondary antibodies. The membranes were developed with the Renaissance chemiluminescence system (PerkinElmer Life Sciences, Inc., Boston, MA, USA). The membranes were placed in an autoradiography cassette, exposed to film and scanned. The primary antibodies used were ATR (Santa Cruz Biotechnology, Dallas, TX, USA, sc-1887), cyclin E (Santa Cruz Biotechnology, sc-240), actin (Millipore, MAB1501R), pCHK1(S345) (Cell Signaling Technology, Danvers, MA, USA, #2348), CHK1 (Santa Cruz Biotechnology, sc-7898), Wee1 (Santa Cruz Biotechnology, sc-5285), pCDK1(Y15) (BD Biosciences #612306), CDK1 (Cell Signaling Technology, #9116), pCDK2(T160) (Cell Signaling Technology, #2561), CDK2 (Santa Cruz Biotechnology, sc-6248), pH2AX(S139) (EMD Millipore, #05-636), PARP (Cell Signaling Technology, #9542), vinculin (Sigma, V9131) and actin (Sigma, St. Louis, MO, USA, Clone C4, #MAB1501R).

### 4.6. ShRNA Knockdown

To generate knockdown cells, shRNA constructs and packaging vectors pMDG.2 and pCMV deltaR8.2 (Didier Trono Laboratory) were co-transfected into HEK293T cells. Lentiviral particles were collected and filtered through 0.44 μm filters. The scrambled lentivirus and vector control lentivirus were generated similarly. The target cells were infected by virus-containing medium as previously described [71]. For cyclin E knockdown cells, the target cells were infected with lentiviral shRNAs for cyclin E and selected and maintained in medium containing 1 μg/mL puromycin. Knockdown of cyclin E was confirmed via immunoblot analysis.

### 4.7. Immunofluorescence Staining

Cells were plated into 8-chamber slides. Following the indicated treatment conditions (main text and figure legends), cells were fixed in 4% paraformaldehyde for 20 min and were permeabilized with a 0.3% TritonX solution (20 mM HEPES, 50 mM NaCl, 3 mM MgCl_2_, 300 mM sucrose, and TritonX-100) for 20 min. Prior to staining with antibodies, nonspecific binding was minimized by blocking with 10% bovine serum albumin and 2% horse serum in phosphate-buffered saline solution (PBS) for 1 h. Cells were then incubated for 1 h with the first antibodies diluted in blocking buffer at room temperature, then treated with secondary goat anti-mouse or goat anti-rabbit antibody (Alexa Fluor 594 or 488 EMD Millipore), diluted at 1:1000 and incubated at room temperature for an additional 1 h. Slides were then mounted with fluorescent mounting medium with DAPI (Dako, Santa Clara, CA, USA). Images were captured by Nikon eclipse 90i microscope and analyzed by ImageJ. For all foci analyses, cells with five or more foci were considered positive. Three biological replicates and three technical replicates were performed for each experiment and more than 200 cells were enumerated for each sample. The primary antibodies for foci staining were: Rad51 (1:5000. Abcam ab63801) and RPA (1:500, calbiochem #NA18).

### 4.8. Cell Cycle Analysis

Flow cytometry was performed as previously described [39]. For apoptotic analysis, for each treatment condition, adherent and floating cells were harvested and combined, washed with PBS and prepared as single-cell suspensions of 1 × 10^6^ cells per mL of PBS. Cells were then fixed with cold 95% ethanol overnight at 4 °C, stained with propidium iodide (PI; Sigma-Aldrich Chemicals) and RNase for 24 h. Cell cycle profiles were generated by a BD Accuri C6 Flow Cytometer. The data were analyzed by FlowJo software (v. 10.0). Samples were also captured on the Beckman Coulter Gallios Flow Cytometer, and data were analyzed with the Kaluza software (Beckman Coulter, Brea, CA, USA).

### 4.9. Annexin/Propidium Iodide Assay

Adherent and floating cells were harvested and combined, washed with PBS and resuspended in 1× Annexin binding buffer, incubated with Alexa488-conjugated Annexin V antibody and PI was used as the live-dead marker. Following incubation for 15 min, cells were diluted in additional 1× binding buffer and analyzed using a BD FACS Calibur flow cytometer in the MD Anderson Flow Cytometry core facility. Results are presented as percentage of cells that were Annexin V positive; cells that were stained with either Annexin V or PI were used as controls to set cell line-specific cut-off values.

### 4.10. Homologous Recombination (HR) Assay

The HR assays were performed as previously described [72]. Briefly, cells were transfected with 3.0 μg (DR-GFP assay) or pCBASceI (the I-SceI expression plasmid). Cells were collected 48 h following transfection, washed twice with PBS, suspended in 0.1% FBS/PBS and fixed with formaldehyde. The proportion of GFP-positive cells was determined using flow cytometry.

### 4.11. Animal Studies

All animal procedures were conducted in compliance with the National Institutes of Health guidelines of animal research and approved by the Institutional Animal Care and Use Committee (IACUC) at the MD Anderson Cancer Center [US Department of Agriculture (USDA) Research Facility Registration #7465, Public Health Service (PHS) Animal Welfare Assurance #A334301, American Association for Accreditation of Laboratory Animal Care (AAALAC) Accredited since 1969]. Six-week-old female BALB/c *nu/nu* mice were obtained from the Department of Experimental Radiation Oncology mouse colony and maintained in the Department of Veterinary Medicine and Surgery at MD Anderson. For SUM149 or HCC1806 cell line xenografts, the mice were injected in the right side of the 4th pair of mammary glands with 4 × 10^6^ cells suspended in 50 μL Dulbecco modified essential medium and 100 μL matrigel (#354234, BD Biosciences). When cell-line xenograft tumors reached 150–200 mm^3^, mice were randomized into 4 treatment arms: (1) Vehicle; (2) AZD-1775 single agent (Wee1i): 50 mg/kg AZD-1775 and vehicle of MK-4827; (3) MK-4827 single agent (PARPi): 50 mg/kg MK-4827 and vehicle of AZD-1775; and (4) combination of AZD-1775 and MK-4827: 50 mg/kg AZD-1775 and 50 mg/kg MK-4827. These treatments were administered one dose per day for 5 days, followed by 2 days without treatment for a 7-day cycle (see Figure S8A for schema). Xenograft SUM149 were treated for 3–4 cycles; xenograft HCC1806 were treated for 4 cycles.

For all mice in all treatment arms, tumor volumes and body weights were measured twice a week. The mice were euthanized when tumor burden reached 1000 mm^3^ or scheduled treatment was completed. Following euthanasia, all tumors were harvested for further analysis. For histology analysis, the tumors were fixed in 4% paraformaldehyde overnight and subjected to paraffin embedding. For Western blot analysis, tumors were snap frozen in liquid nitrogen within 30 min of excision and stored at −80 °C.

### 4.12. RNA Sequencing and Gene Set Enrichment Analysis (GSEA)

MDA231 cells were treated with (i) vehicle, (ii) 5 μM MK-4827 (PARPi), (iii) 2.5 μM AZD-1775 (Wee1i) and (iv) combination of MK-4827 and AZD-1775 for 48 h and cultured in drug free media for an additional 48 h, harvested and total RNA from each sample was isolated using a RNeasy Kit with DNase treatment (#74104 and #79254, Qiagen, Germantown, MD, USA). RNA samples were submitted to the Sequencing and Microarray Core Facility at MD Anderson Cancer Center. mRNAs were enriched by Poly(A) selection, and the libraries were prepared by mRNA-Seq Sample Prep Kit (Illumina, Foster City, CA, USA) following the manufacturer’s instructions. Pair-end 76-bp sequencing was performed by Illumina HiSeq 2000. FASTQ sequence files were obtained, and the RNA-seq reads were aligned to the human reference genome. Functional analysis of the differentially expressed transcripts was performed using gene set enrichment analysis (GSEA) as described [73].

### 4.13. Statistical Analysis

Breast cancer cases from the TCGA and METABRIC database were analyzed using the Cbioportal website (www.cbioportal.org, accessed on 25 September 2017. Wilcoxon rank sum test was used for the cyclin E level comparison between the wild-type and mutation cohorts. The distribution of cyclin E DNA/RNA alteration was compared by Chi-squared test. The differences in overall survival curves were evaluated by log-rank test.

Each cell culture experiment had at least three technical and three biological repeats. Continuous outcomes were summarized with means and standard errors. Comparisons between two groups were analyzed by two-sided *t*-tests using MS Excel or Prism 8. Comparisons among groups of more than 2 were analyzed by one-way ANOVA using Prism 8. Tumor volume of each mouse was normalized to its volume on the first day of treatment (day 1). On day 22, the normalized tumor volume was compared by two-sided *t*-tests, using MS Excel. The survival time/percent for each mouse was calculated when the maximum tumor burden (1000 mm^3^) was reached or the mouse died due to other reasons. The overall Kaplan–Meier survival curves in the mouse studies were generated by Prism 8 software (GraphPad), and the significance was determined using a log-rank (Mantel–Cox) test. All graphs were generated either in MS Excel or Prism 8.

## Data Availability

The data presented in this study are available in this article (and Supplementary Materials).

## Supplementary Materials

The following are available online at https://www.mdpi.com/article/10.3390/cancers13071656/s1, Figure S1: Cyclin E1 alteration is enriched in BRCA1/2 mutated breast cancers, Figure S2: Cyclin E overexpression sensitizes cells to PARP inhibition, Figure S3: Combination treatment of PARPi and Wee1i in breast cancer, Figure S4: Combination treatment of PARPi and Wee1i accelerates DNA replication stress, Figure S5: Combination treatment of PARPi and Wee1i deregulates the cell cycle, Figure S6: GSEA analysis of DNA repair pathways, Figure S7: Combination treatment of PARPi and Wee1i accelerates DNA damage, Figure S8: Combination treatment of PARPi (MK-4827) and Wee1i (AZD-1775) is synergistic in vivo.
