# Supplementary material for: Targeting Replicative Stress and DNA Repair by Combining PARP and Wee1 Kinase Inhibitors Is Synergistic in Triple Negative Breast Cancers with Cyclin E or BRCA1 Alteration
Source: Cancers (Basel). 2021 Apr 1;13(7):1656. doi: 10.3390/cancers13071656 (PMC8036262; doi:10.3390/cancers13071656)
Supplement: Supplementary file 1 [file cancers-13-01656-s001.zip › cancers-1135759-supplementary-for xml/cancers-1135759-supplementary-for xml.docx]

Supplementary Materials: Targeting Replicative Stress and DNA Repair by Combining PARP and Wee1 Kinase Inhibitors is Synergistic in Triple Negative Breast Cancers with Cyclin E or *BRCA1* Alteration

Xian Chen, Dong Yang, Jason P. W. Carey, Cansu Karakas, Constance Albarracin, Aysegul A. Sahin, Banu K. Arun, Merih Guray Durak, Mi Li, Mehrnoosh Kohansal, Tuyen N. Bui, Min Jin Ha, Kelly K. Hunt and
Khandan Keyomarsi


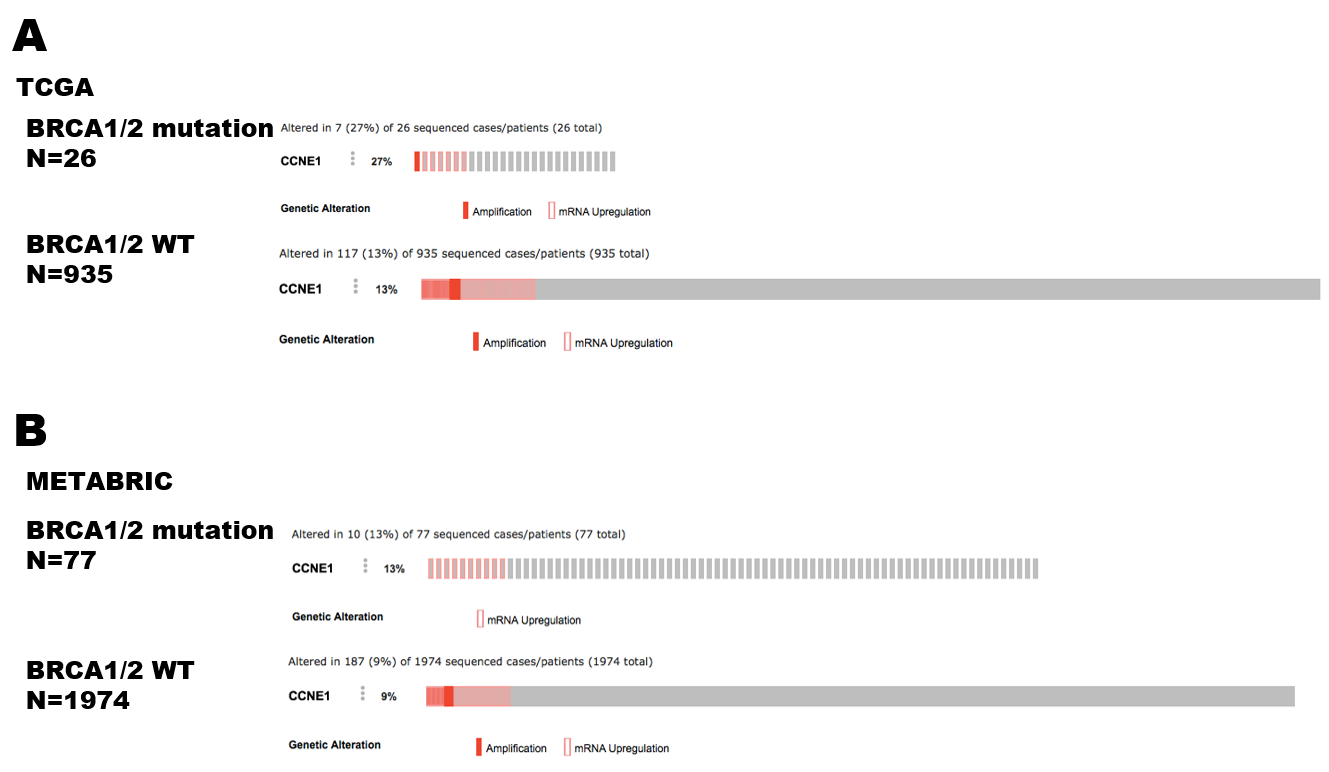


**Figure S1.** Cyclin E1 alteration is enriched in *BRCA1/2* mutated breast cancers. Percentages of *BRCA1/2* mutated and WT breast tumors with Cyclin E1 alteration from the TCGA **(A)** and METABRIC **(B)** database ([www.cbioportal.org](http://www.cbioportal.org)).


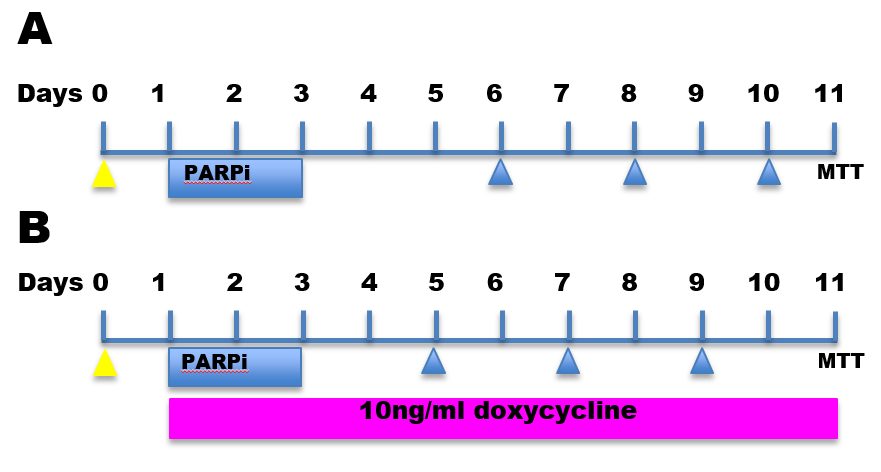

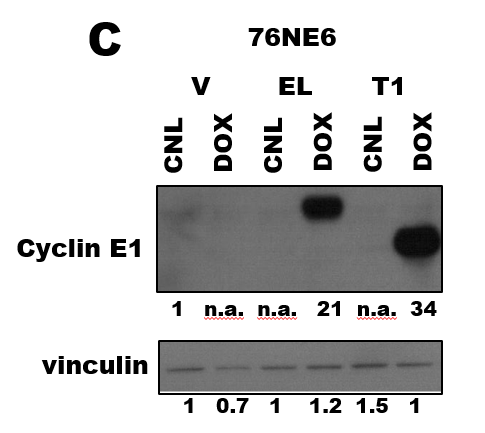


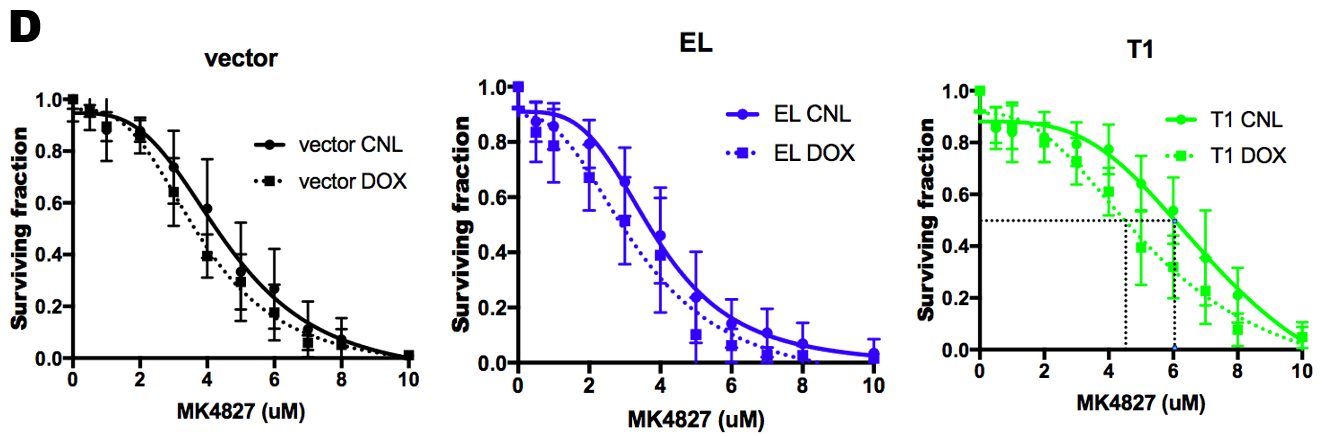


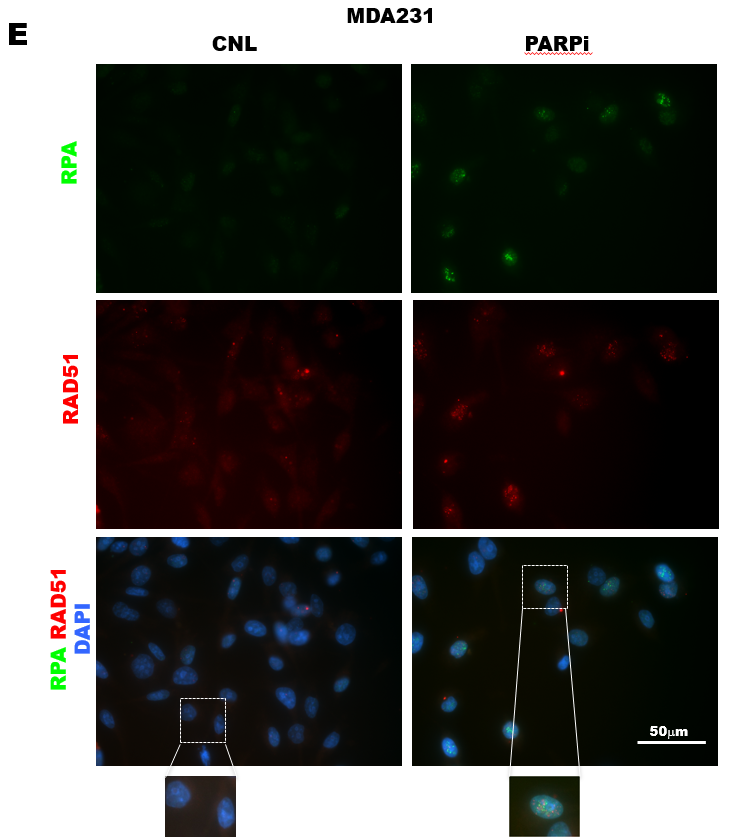


**Figure S2.** Cyclin E overexpression sensitizes cells to PARP inhibition. (**A**) Schematic of high through put survival assay (HTSA) treatment strategy using PARPi (MK-4827). Yellow arrowhead indicate time when cells were seeded; Blue Box indicates the duration of treatment with PARPi (MK-4827); blue arrowheads indicate days when cultures were refreshed with drug free media changing. (**B**) Schematic of HTSA with PARPi (MK-4827) treatment and doxycycline induction. Seeding of cells, treatment with PARPi (MK-4827) and drug free media changes are as in A. Pink box indicates duration of doxycycline treatment. (**C**) immunoblot of cyclin E in 76NE6 cells transfected with empty vector, cyclin E-inducible (EL) or LMWE-inducible (T1) vectors treated with (DOX) and without (CNL) doxycycline for 24 hours. Densitometry was performed on all western blots and the relative expression of each band to its loading control in noted on the bottom of each panel for each antibody used. n.a. densitometry is not available because the western blot band cannot be detected (**D**) HTSA of 76NE6 cells with vector (left), cyclin E-inducible (EL, middle) or LMWE-inducible (T1, right) vectors as in **B**. (**E**) Representative RPA and RAD51 foci in MDA231 cells treated with 5 µM PARPi (MK-4827) or without (CNL) 48 hours. The areas inside the white box are shown in Figure 2G and enlarged on the bottom of each panel. Scale bar, 50 µm. Error bars represent standard error of the mean.


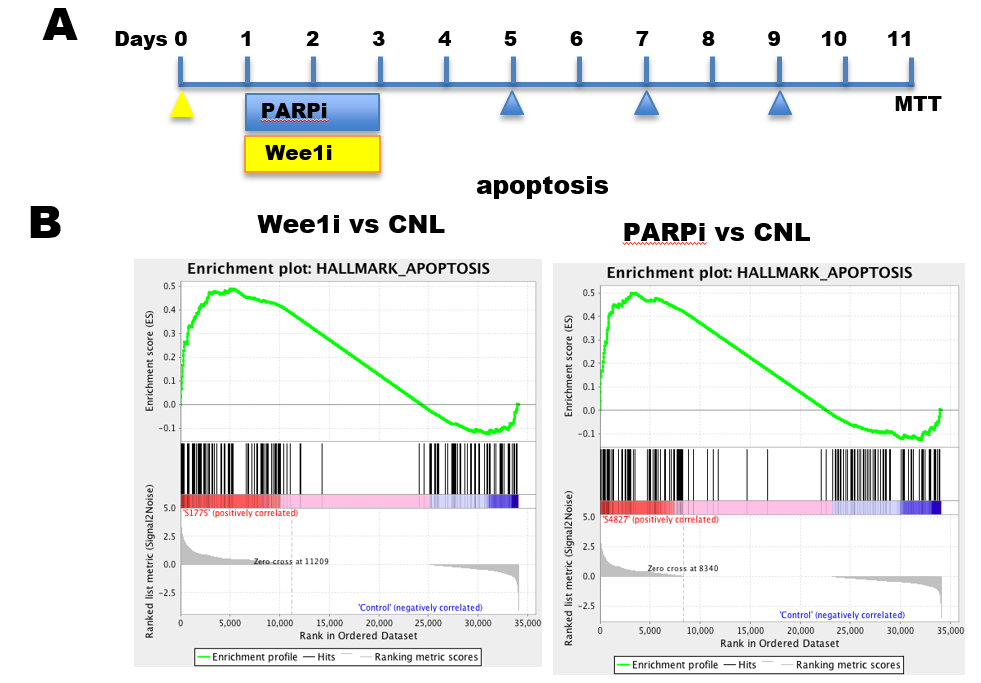


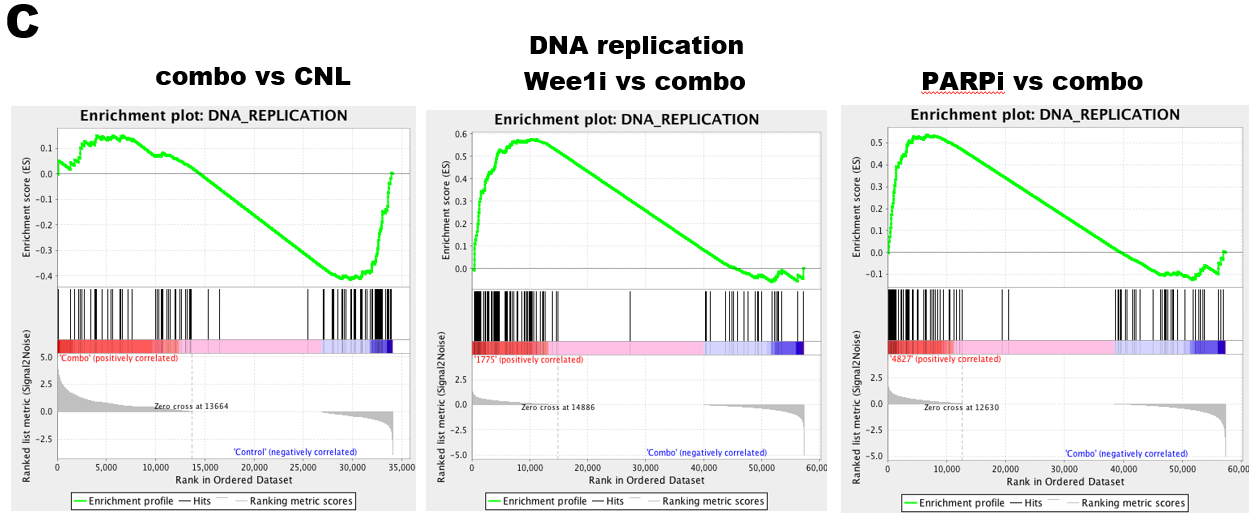


**Figure S3.** Combination treatment of PARPi and Wee1i in breast cancer. (**A**) Schematic of HTSA of combination treatment with PARP (MK-4827) and Wee1i (AZD1775). Yellow arrowhead indicate time when cells were seeded; Blue and Yellow Boxes indicate the duration of treatment with PARPi (MK-4827) or Wee1i (AZD1775) treatment, respectively and blue arrowheads indicate days when cultures were refreshed with, drug free media media (**B)** The apoptosis gene set enrichment and **(C)** the DNA replication gene set enrichment by the GSEA analysis of the RNA sequencing result of MDA231 show (i) apoptosis pathway is enriched in Wee1i and PARPi treated cells as compared to control and (ii) DNA replication pathway is enriched in the control, Wee1i (AZD-1775) or PARPi (MK-4-827) single arm treated groups as compared to combo treated cells.


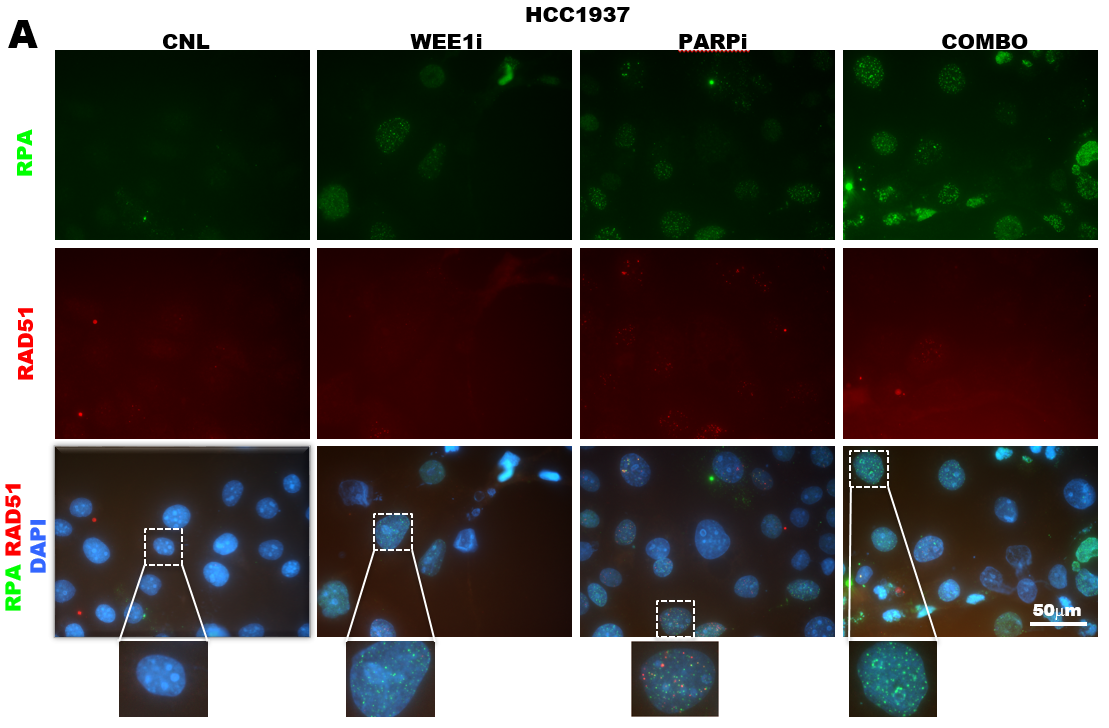


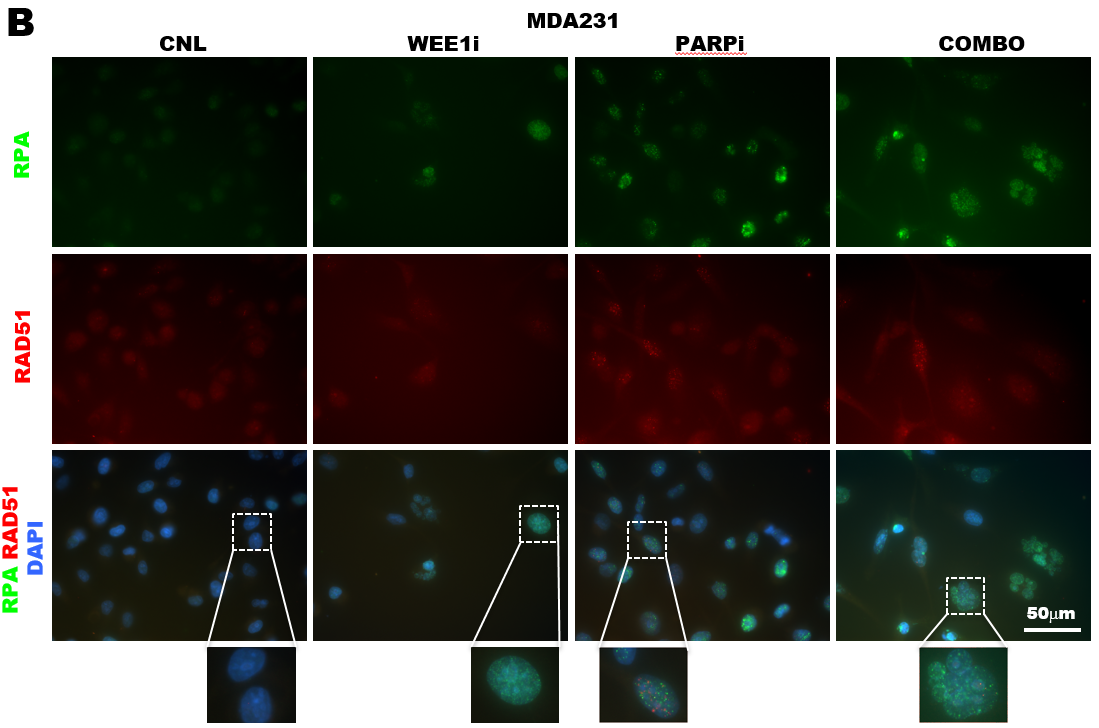


**Figure S4.** Combination treatment of PARPi and Wee1i accelerates DNA replication stress. Representative images of RPA (green) and RAD51 (red) foci of (**A**) HCC1937 and (**B**) MDA231 cells treated with vehicle control; Wee1i, AZD1775 (2.5 μM for MDA231 and 0.15 μM for HCC1937); PARPi, 5 μM MK4827; COMBO, the combination treatment of AZD1775 and MK4827 as the same dosage of mono-treatment, all for 48 hours. The areas inside the white box are shown in **Figure 4A** and enlarged on the bottom of each panel.


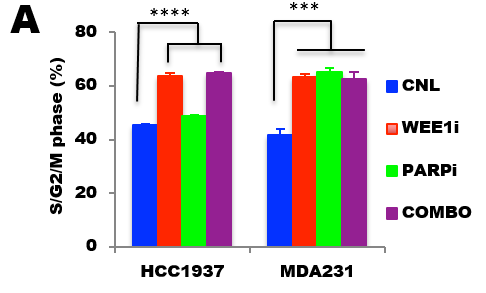

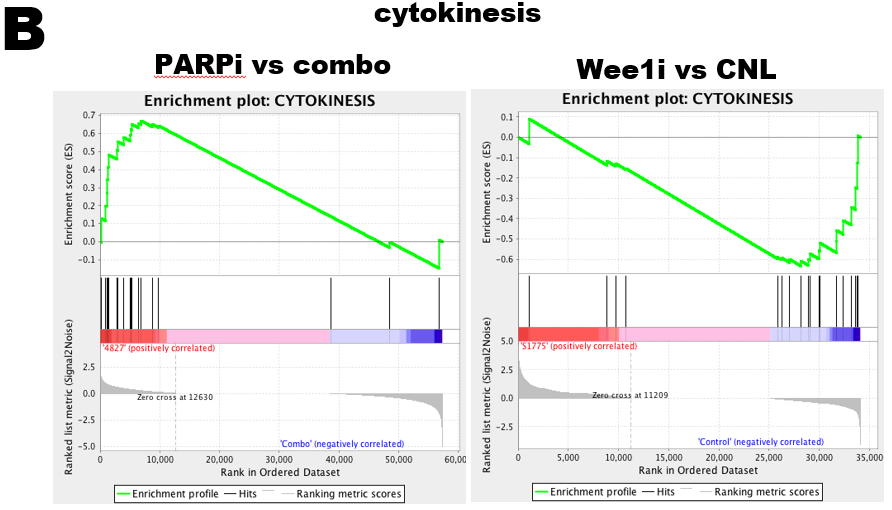


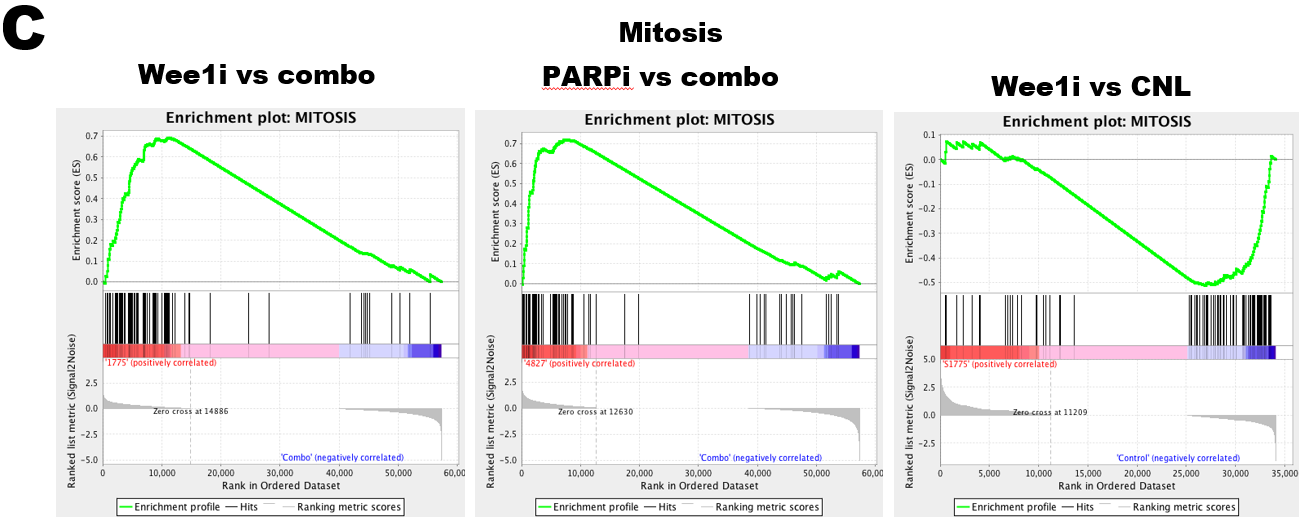


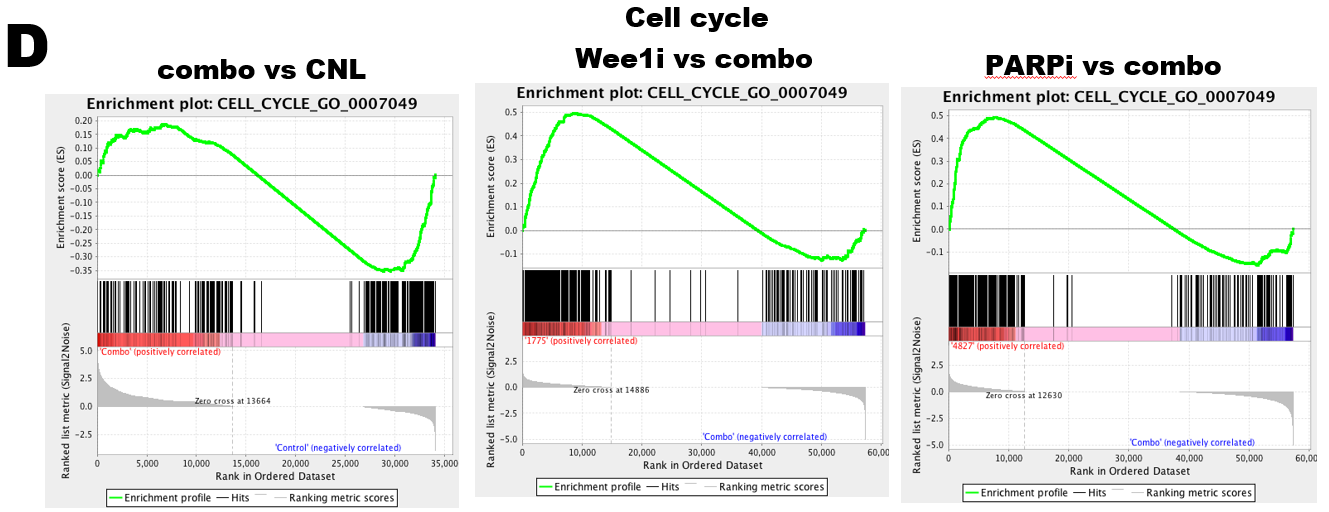


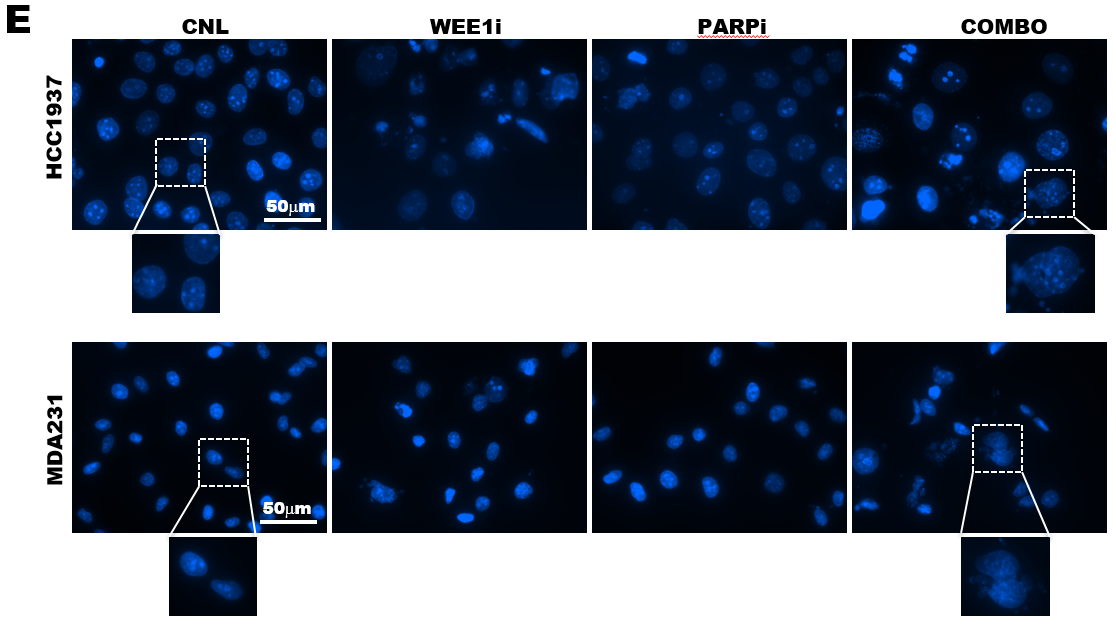


**Figure S5.** Combination treatment of PARPi and Wee1i deregulates the cell cycle. (**A**) Cell cycle (S, G2 and M phases) analysis quantitated using flow cytometry following 48 hours treatment with vehicle control; Wee1i, AZD1775 (2.5 μM for MDA231 and 0.15 μM for HCC1937); PARPi, 5 μM MK4827; COMBO, the combination treatment of AZD1775 and MK4827 as the same dosage of mono-treatment. Gene set enrichment analysis for (**B)** the cytokinesis **(C)** the mitosis and **(D)** the cell cycle gene sets using the RNA sequencing results of MDA231 cells. These GSEA analyses show cytokinesis enriched in PARPi (MK-4827) as compared to combo and in control (CNL) as compared to Wee1i (AZD-1775) treated arms. Mitosis is enriched in Wee1i (AZD-1775) and PARPi (MK-4827) single arm treated cells as compared to combo and in control (CNL). Cell cycle GSEA analysis shows enrichment in the control, Wee1i (AZD-1775) and PARPi (MK-4827) treated cells as compared to the combo treatment. **(E)** Representative images of 4',6-diamidino-2-phenylindole (DAPI) staining of HCC1937 (top) and MDA231 (bottom) cells treated with the 48-hour treatment as in **A**. Scale bar, 50 µm. The areas inside the white box are shown in Figure **4G** and **4H** and enlarged on the bottom of each panel.


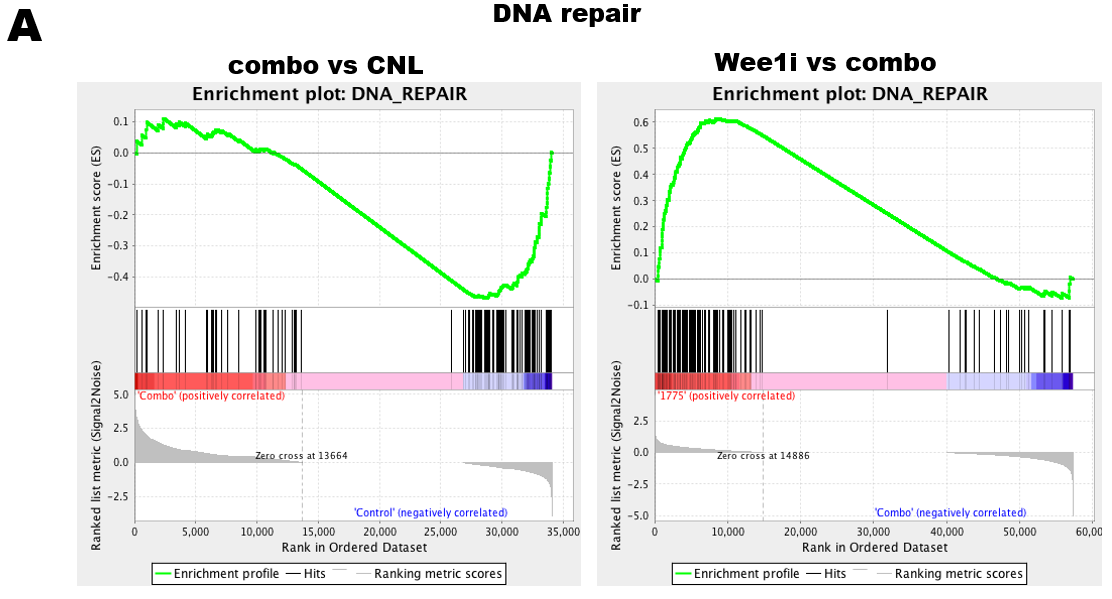


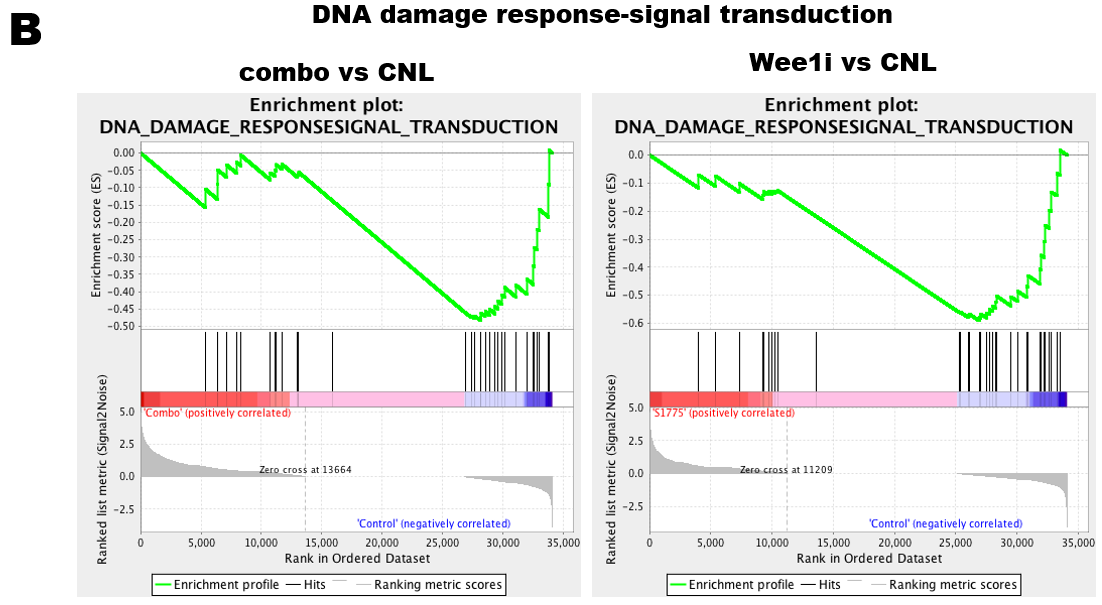


**Figure S6.** GSEA analysis of DNA repair pathways. Gene set enrichment analysis (GSEA) for (**A)** DNA repair and **(B)** DNA damage response-signal transduction gene sets using the RNA sequencing results of MDA231 cells (Figure **3B)**. These GSEA analyses show DNA repair pathway is enriched in control (CNL) and Wee1i (AZD-1775) treated cells as compared to combo. DNA damage response-signal transduction shows enrichment of this gene set in control (CNL) as compared to combo and Wee1i (AZD-1775) treated cells.


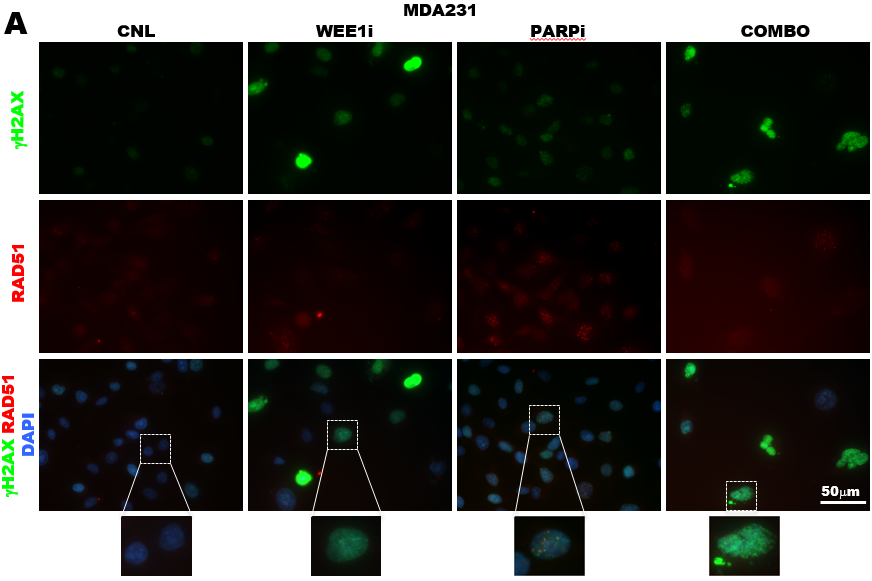


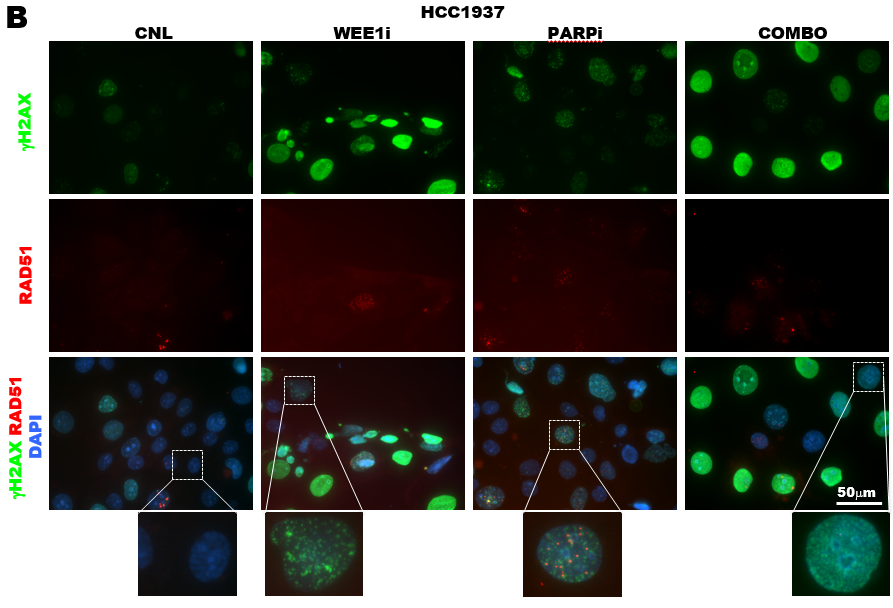


**Figure S7.** Combination treatment of PARPi and Wee1i accelerates DNA damage. Representative images of γH2AX (green) and RAD51 (red) foci of (**A**) MDA231 and (**B**) HCC1937 cells following 48 hours treatment with vehicle control; Wee1i, AZD1775 (2.5 μM for MDA231 and 0.15 μM for HCC1937); PARPi, 5 μM MK4827; COMBO, the combination treatment of AZD1775 and MK4827 as the same dosage of mono-treatment. Areas inside the white box are shown in Figure **5B** and **5C** also enlarged at the bottom of each panel.


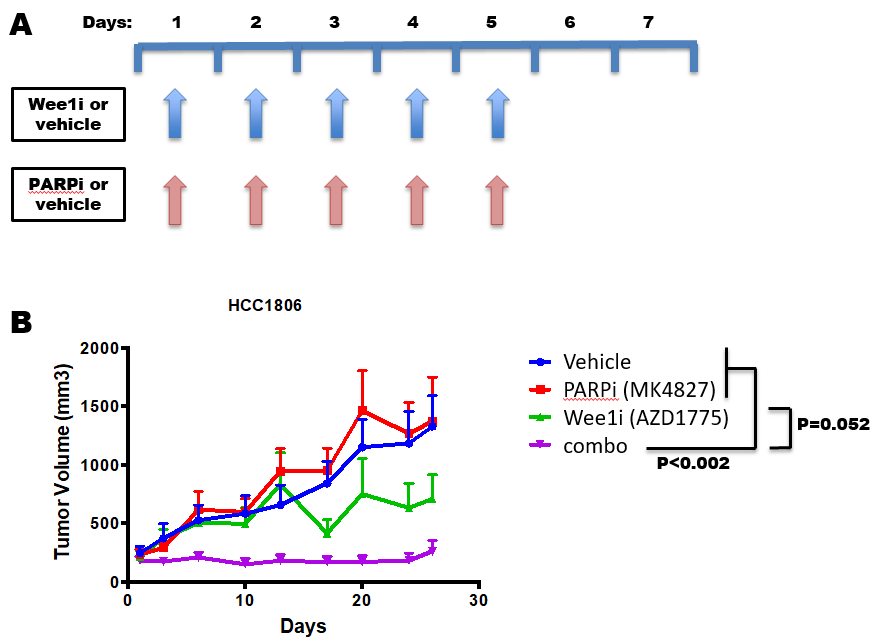


**Figure S8.** Combination treatment with PARPi (MK-4827) and Wee1i (AZD-1775) is synergistic *in vivo*. (**A**) Schematic of 1 cycle of the combination treatments of PARPi (MK-4827) and Wee1i (AZD1775) *in vivo*. 50 mg/kg MK4827 (red arrow) and/or 50 mg/kg AZD1775 (blue arrow) was administered via oral gavage once per day for 5 days per week. (**B**) Tumor volumes of mice harboring HCC1806 cells xenograft tumors were treated as in A for 4 cycles. The multiple comparison of one-way ANOVA was used to compare tumor weights. Error bars represent standard error of the mean.
