# Supplementary material for: Targeting Replicative Stress and DNA Repair by Combining PARP and Wee1 Kinase Inhibitors Is Synergistic in Triple Negative Breast Cancers with Cyclin E or BRCA1 Alteration
Source: Cancers (Basel). 2021 Apr 1;13(7):1656. doi: 10.3390/cancers13071656 (PMC8036262; doi:10.3390/cancers13071656)

Uncropped Western Blots

Targeting Replicative Stress and DNA Repair by Combining PARP and Wee1 Kinase Inhibitors is Synergistic in Triple Negative Breast Cancers with Cyclin E or *BRCA1* Alteration

Authors: Xian Chen, Dong Yang, Jason P. W. Carey, Cansu Karakas, Constance Albarracin, Aysegul A. Sahin, Banu K. Arun, Merih Guray Durak, Mi Li, Mehrnoosh Kohansal, Tuyen N. Bui, Min Jin Ha, Kelly K. Hunt and Khandan Keyomarsi

Figure 2B

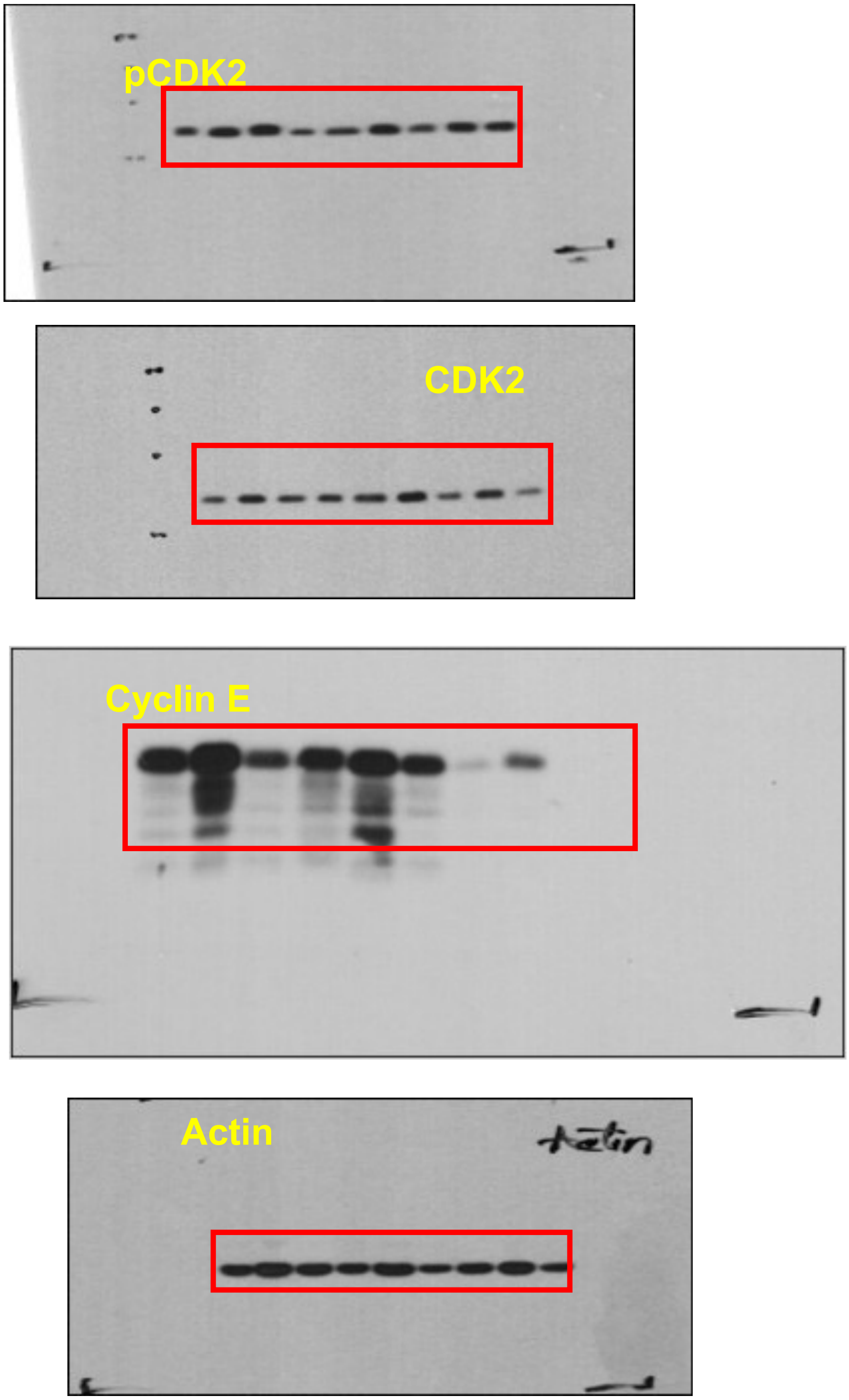

Figure 2E

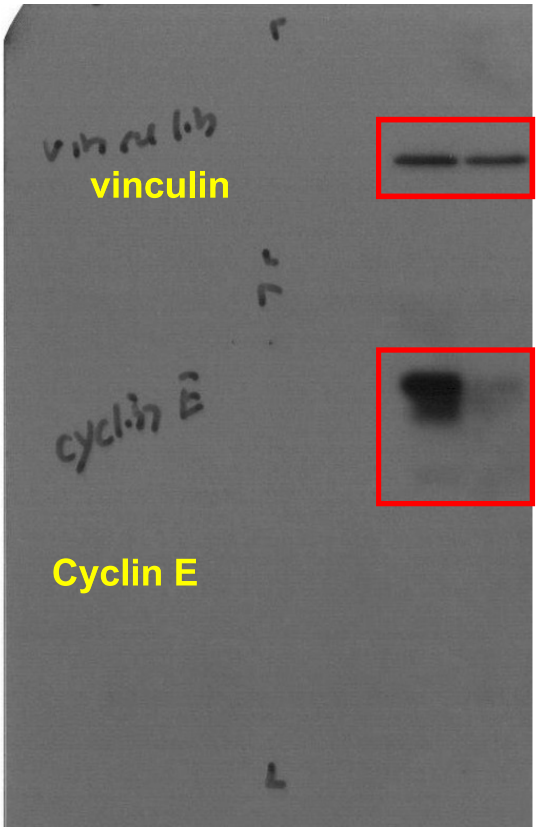

Figure 3D

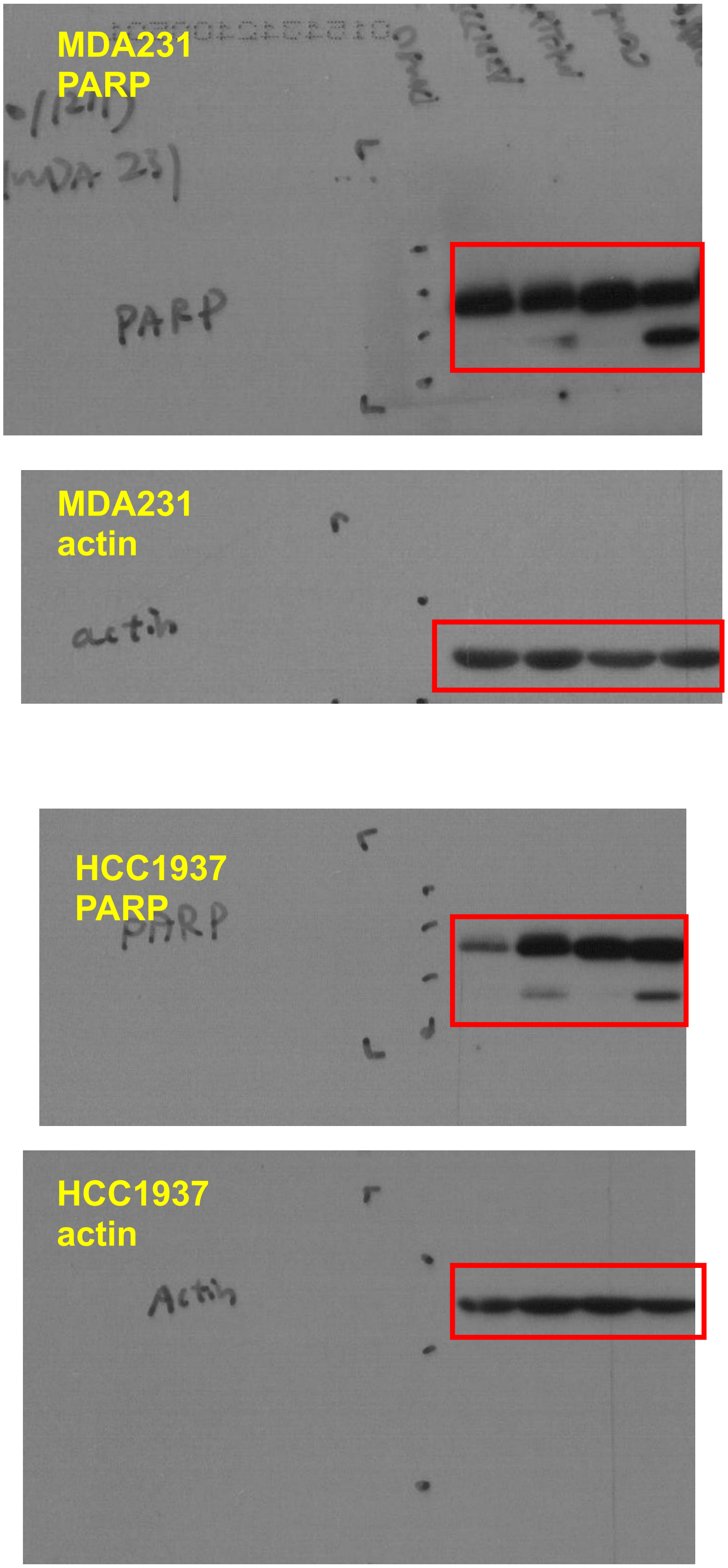

Figure 4D (MDA231)

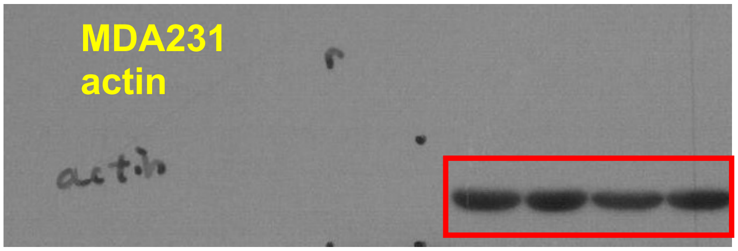

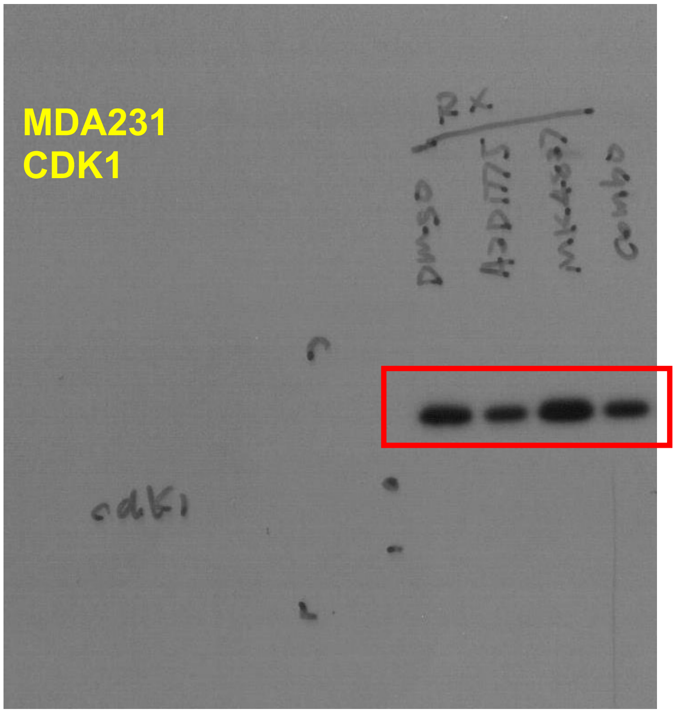

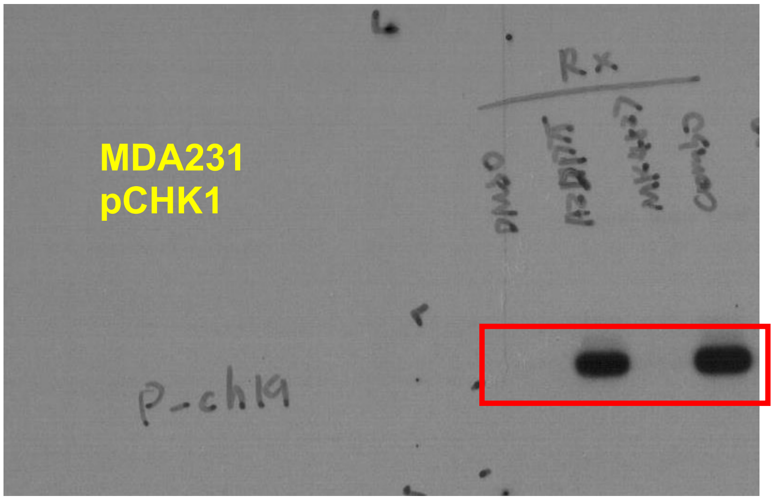

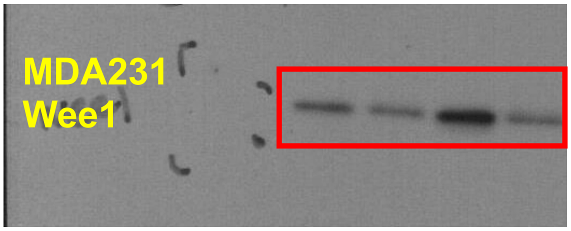

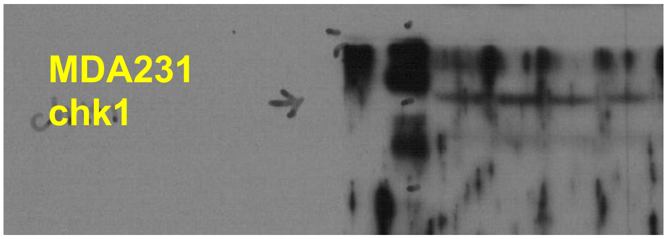

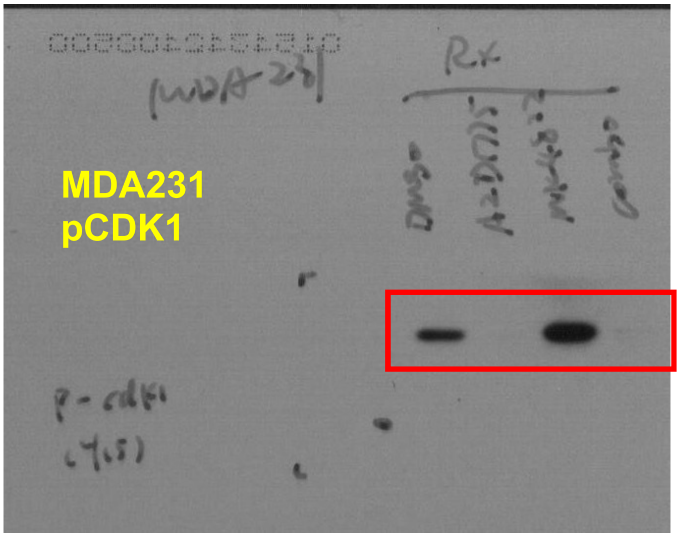

Figure 4D (HCC1937)

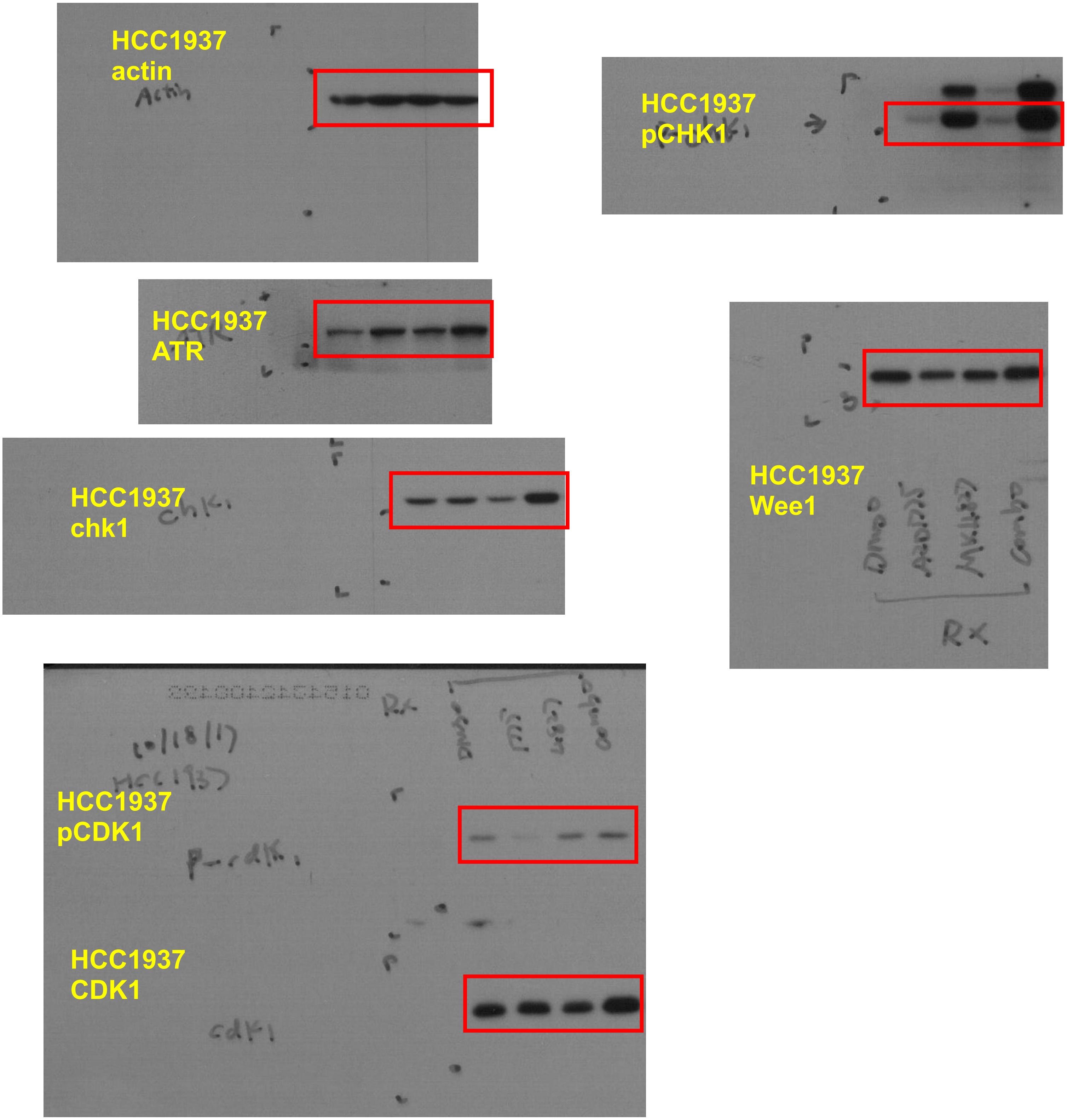

Figure 5C (MDA231)

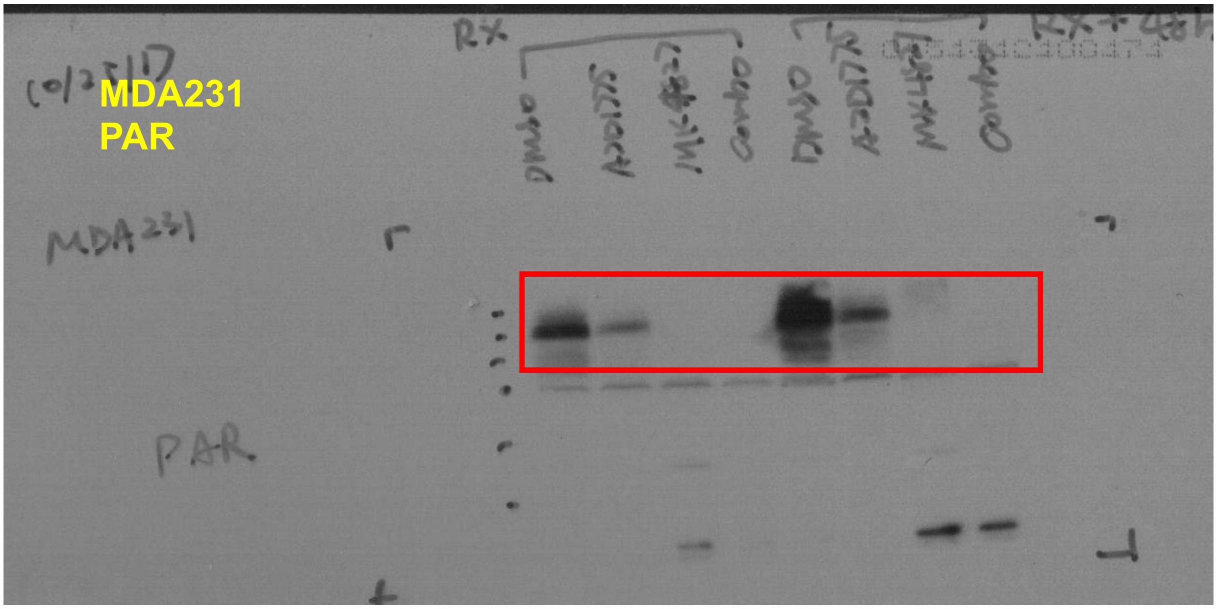

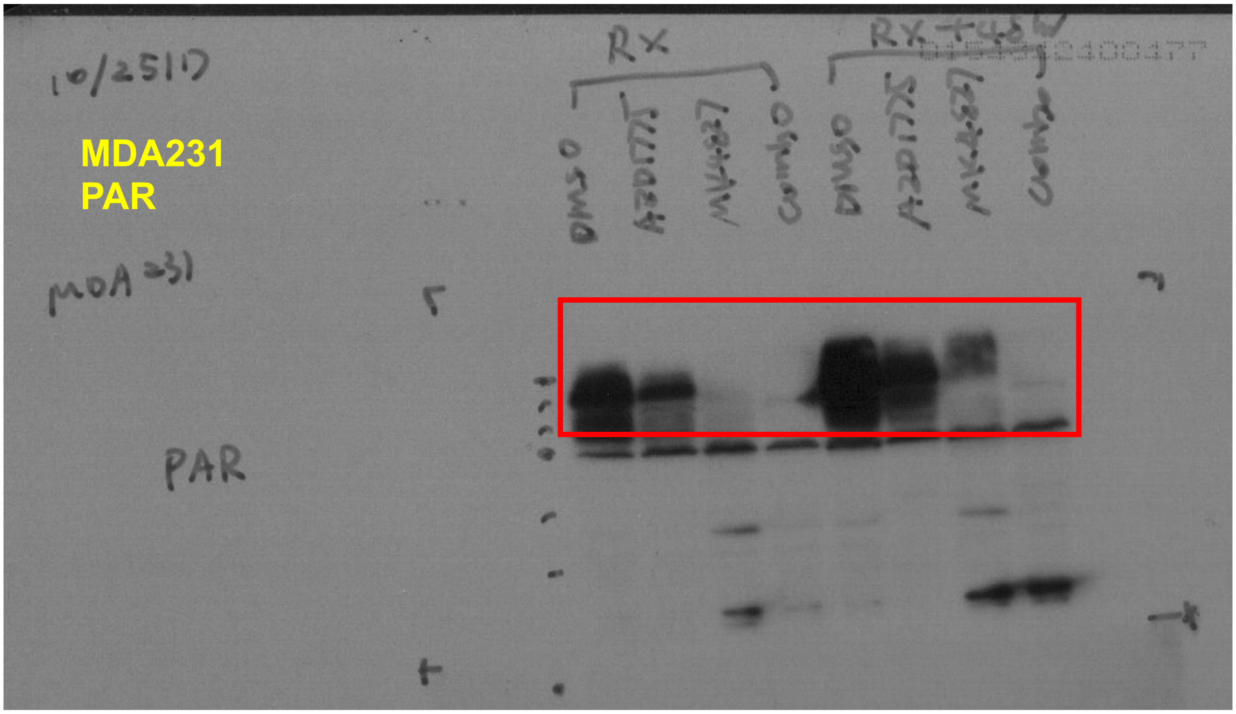

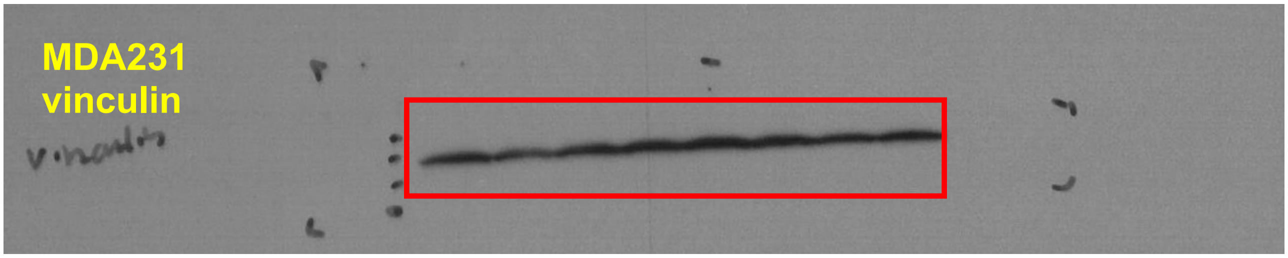

Figure 5C (HCC1937)

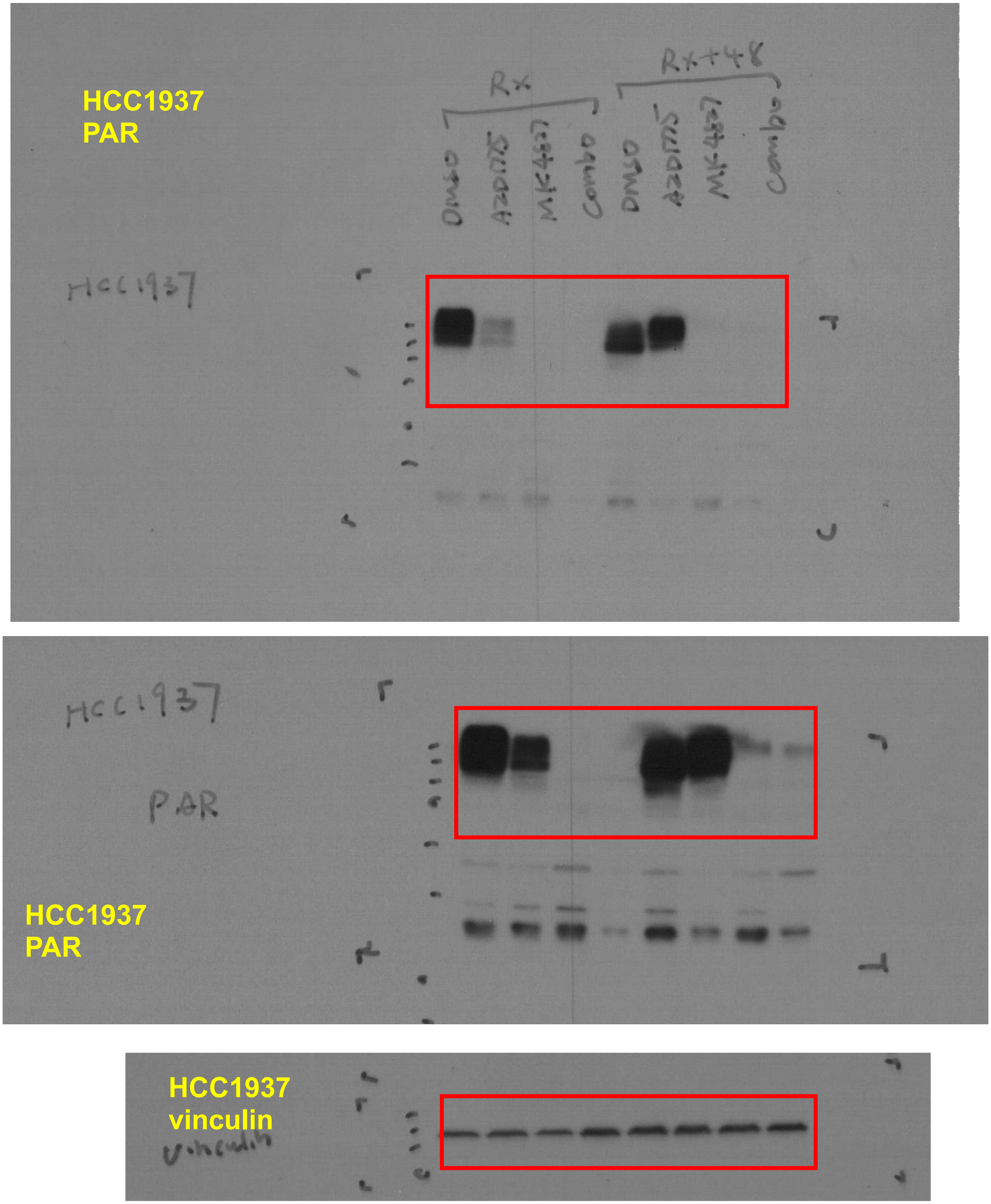

Figure 5F

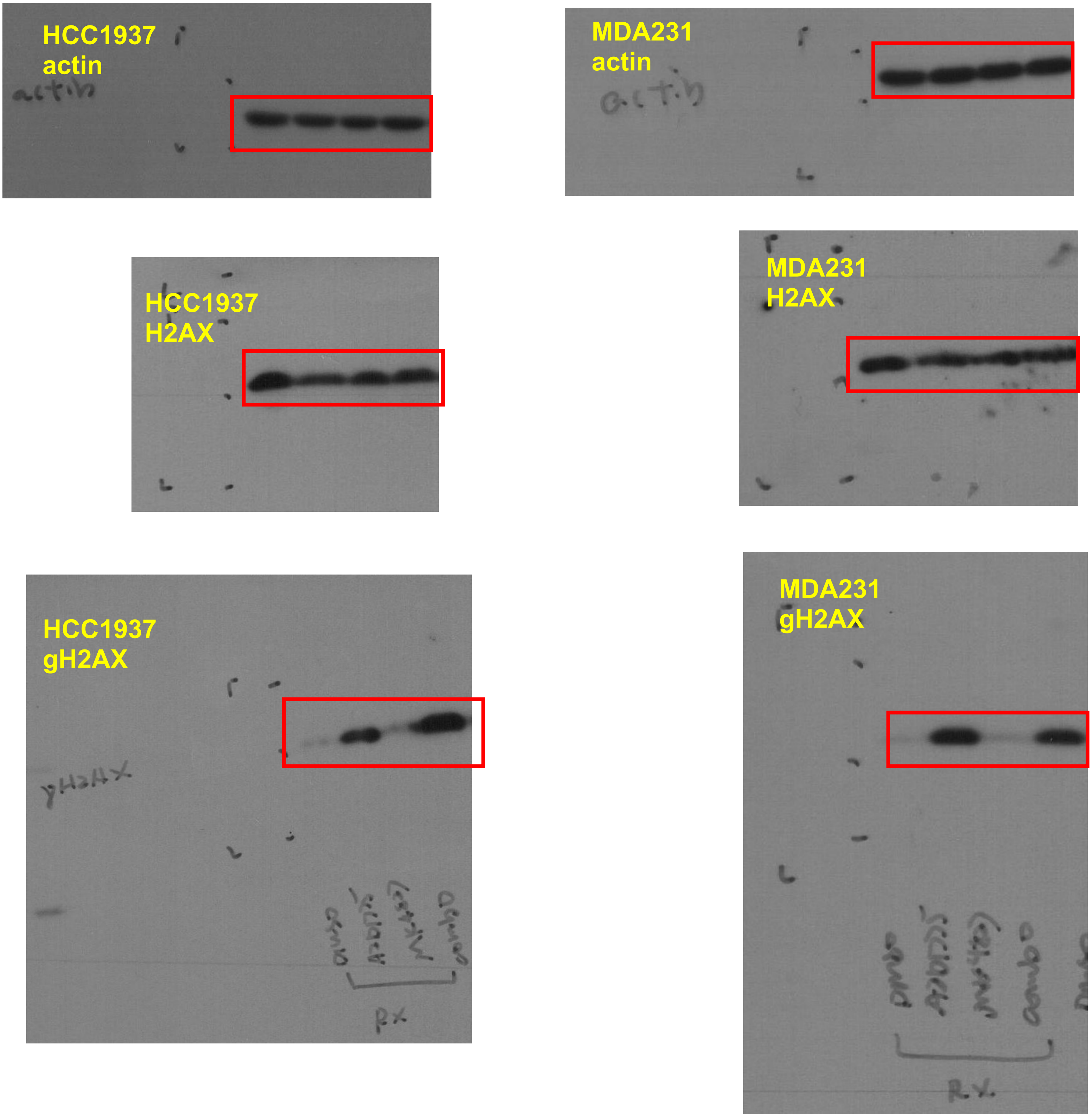
HCC1937
actin
MDA231
actin
HCC1937
H2AX
MDA231
H2AX
HCC1937
gH2AX
MDA231
gH2AX

Figure 6H

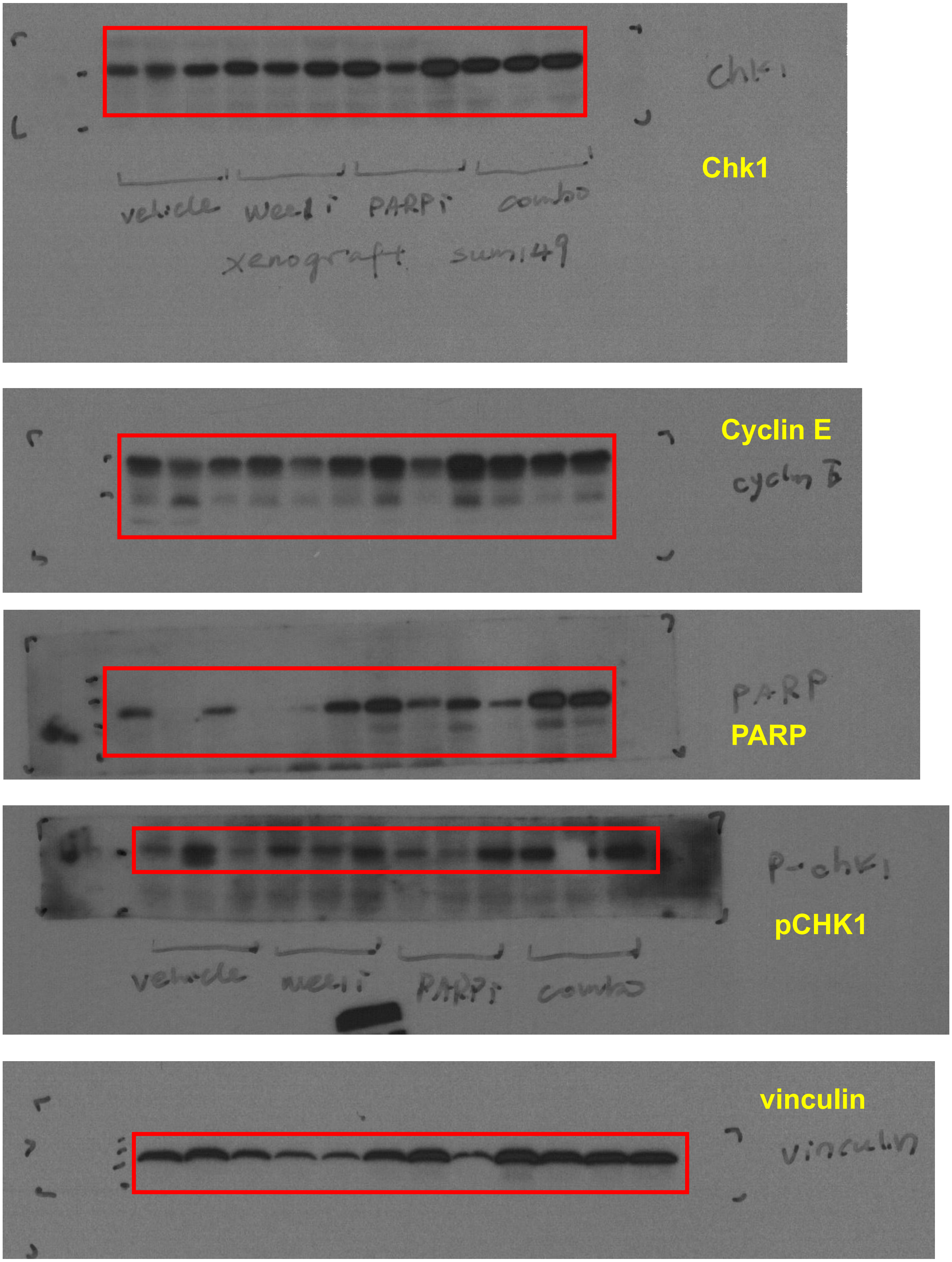

Figure S2C

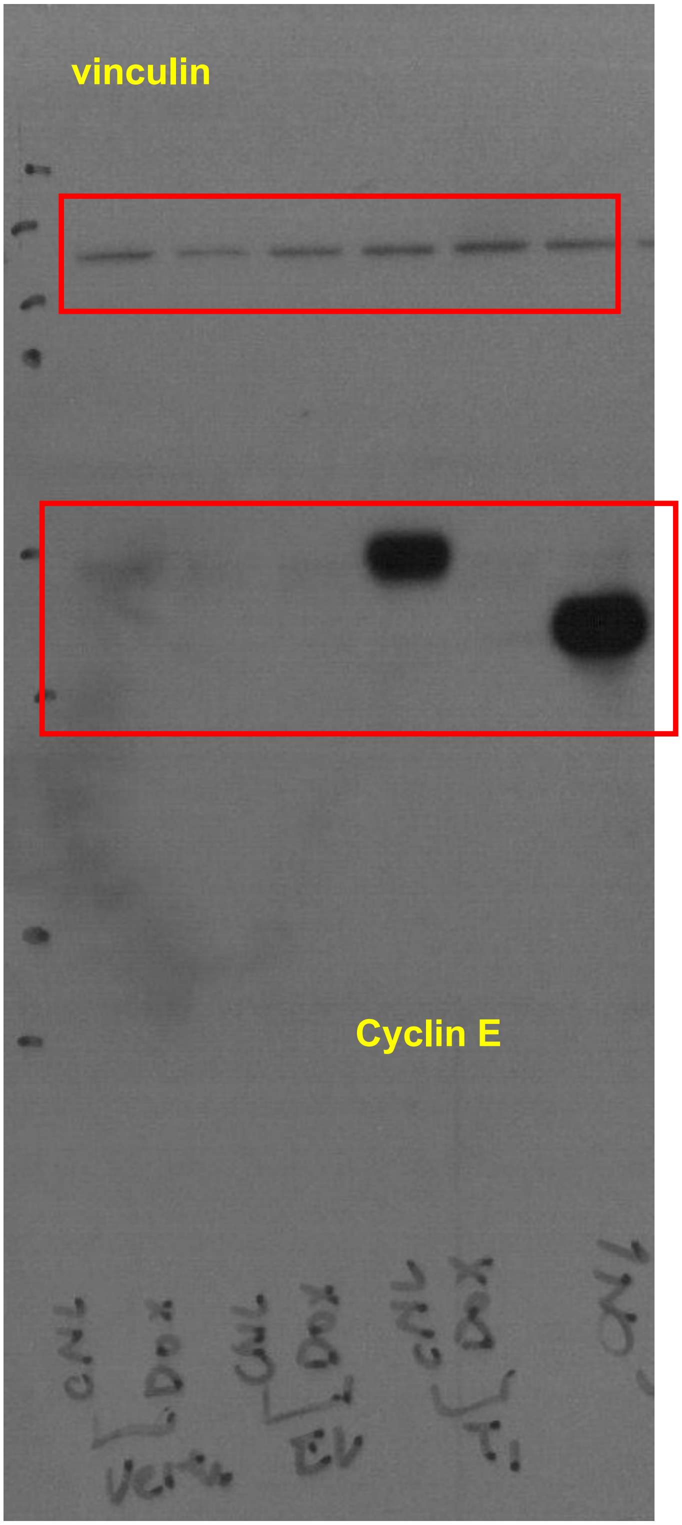

Supplement: Supplementary file 1 [file cancers-13-01656-s001.zip › cancers-1135759-supplementary-for xml/cancers-1135759-WB figures.pdf]
